# Shapeshifting Liquid Metal Droplets for Soft Fluidic Machines

**DOI:** 10.1002/adma.202420265

**Published:** 2025-10-23

**Authors:** Saba Firouznia, Christian Romero, Hemma Philamore, Andrew Conn, Martin Garrad, Jonathan Rossiter

**Affiliations:** ^1^ School of Engineering Mathematics and Technology University of Bristol Bristol BS8 1TW UK; ^2^ Bristol Robotics Laboratory Bristol BS16 1QY UK

**Keywords:** adaptive functional materials, droplet manipulation, fluidic energy transduction, liquid metal actuator, soft matter systems

## Abstract

Studying droplet dynamics is crucial for microfluidics, materials science and soft robotics. Electric and magnetic fields enable precise droplet handling for drug delivery and diagnostics, but high‐energy inputs trigger droplet instabilities and explosive breakup. Here these materials instabilities are leveraged for high energy transduction in liquid metal droplets with high surface tension and conductivity. Liquid metal shapeshifting (LMSS) is introduced, a method for fluidic power generation that uses Lorentz forces and surface tension. Demonstrated in a low voltage (mean≤0.1V), soft, bidirectional pump, it features a simple design with two electrodes, one liquid metal drop, and a small magnet, which can be driven directly from an AAA battery, outperforming previous pumps. Demonstrating versatility, this shapeshifting approach is applied to create an active wearable photoprotective skin, soft robotic actuators, and adaptive color‐changing units for clothing, establishing a range of material‐focused applications. The liquid metal shapeshifting system represents a transformative material platform for compact, integrated fluidic power supplies and its potential spans applications across lab‐on‐a‐chip devices, micro/macro robotics, adaptive materials, and wearable technologies, positioning it as a foundational technology for the next generation of multifunctional, fluidic‐driven soft machines.

## Graphical abstract

This study presented a liquid metal shape shifting strategy which harnesses Lorentz force, surface tension and fluid instabilities to achieve autonomous fluidic power. By using a liquid metal droplet as a mobile current carrier, this strategy enables the development of low‐voltage, self‐oscillating pumps that outperform existing soft pumps. The liquid metal shapeshifting principle has broad applications in micro/macro robotics, microfluidics, lab‐on‐a‐chip devices, and adaptive materials, paving the way for next generation multifunctional fluidic‐driven soft machines.

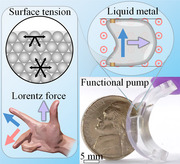

## 1 Introduction

The manipulation of droplets is central to many applications, including advanced materials, drug delivery, bioassays, and chemical reactions,^[^ ^1^, ^2^ ^]^ where external fields including electric, magnetic, acoustic, and light enable precise droplet control.^[^ ^2^, ^3^, ^4^, ^5^ ^]^ However, as input energies increase, the control and scaling of such system becomes challenging as critical material instabilities are reached, and droplets catastrophically break up. If these instabilities, and critical aspects of separation and coalescence,^[^ ^6^ ^]^ could be controlled and harnessed, high energy transduction systems could be realized. The potential of high energy droplet manipulation is important for the development many fluidic devices including soft actuators, where fluidic principles are used to induce forces and deformations in flexible materials. Understanding and exploiting droplet dynamics is particularly important for liquid metal droplets which exhibit extremely high surface tension and excellent conductivity,^[^ ^6^, ^7^, ^8^, ^9^, ^10^ ^]^ enabling them to effectively manage substantial mechanical, thermal, and electrical energy.^[^ ^11^, ^12^, ^13^ ^]^

**Figure 1:**
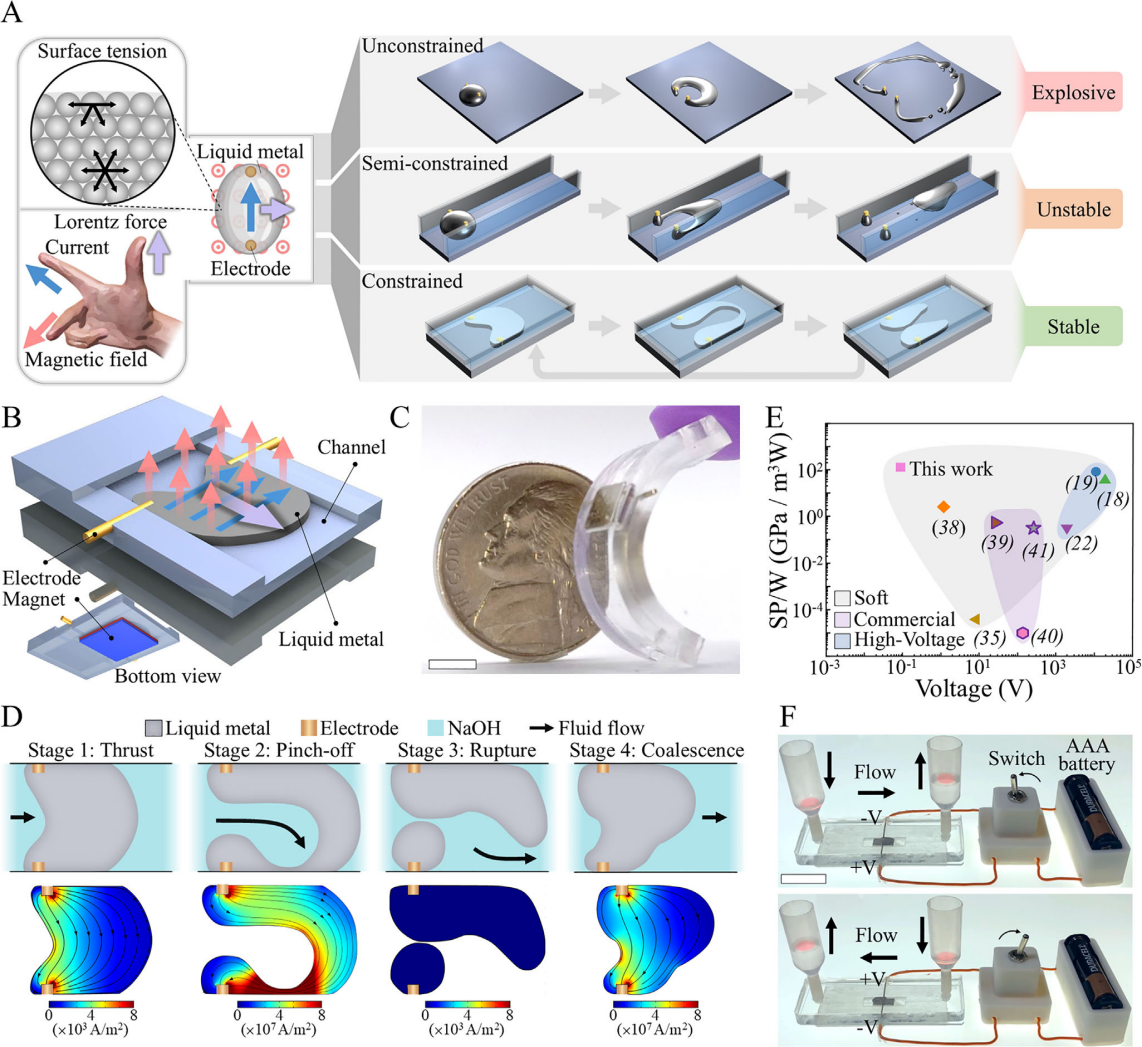
The concept of liquid metal shapeshifting. A) Conceptual figure of the liquid metal shapeshifting droplet in unconstrained (explosive), semi‐constrained (unstable) and constrained (stable, cyclic) conditions, interplaying intrinsic surface tension and the Lorentz force. B) Schematic diagram of the LMSS pump. Current, magnetic field and Lorentz force are shown as blue, red and purple arrows. C) Demonstrating the deformability and small size of the LMSS pump (scale bar 5 mm). D) (top) 4 stages of the liquid metal shapeshifting in the LMSS pump: 1‐deforming, 2‐pinch‐off, 3‐rupture, 4‐coalescence and return to stage 1. (bottom) Simulation of the current density of the liquid metal at each stage. E) Comparison of specific pressure per Watt versus operating voltage of LMSS pump and other soft and commercial pumps. The detailed data are presented in Table S (Supporting Information). F) Demonstrating bidirectionality of LMSS pump driven directly from an AAA battery. Scale bar is 20 mm.

While it is common to avoid fluidic instability due to its unpredictability and dynamic nature,^[^ ^7^ ^]^ our work focuses on harnessing and controlling these instabilities to create autonomous cyclic droplet motion, delivering simple, high energy and sustainable energy transduction. To achieve this, we explore the interplay of energy input, droplet stability and boundary constraints. In this study, we combine the self‐morphing, high conductivity and fluidic hysteresis of liquid metal droplets to demonstrate liquid metal shapeshifting (LMSS), a transduction concept which can power the next generation of fluidic devices. Here, unlike previous liquid metal shape manipulation mechanisms,^[^ ^14^, ^15^, ^16^ ^]^ we harness the multiphysics interaction of non‐stationary Lorentz forces (established in the liquid metal, which acts as a mobile current carrier) and the surface tension of the liquid metal (which controls liquid metal breakup and coalescence)^[^ ^17^ ^]^ to generate an autonomous fluid motor effect. In the simplest embodiment, if we pass a current through liquid metal in a magnetic field, it will stretch and morph away from the electrodes due to induced Lorentz forces (**Figure** **1**A left, Video S1, Supporting Information), storing surface energy as its area expands. If unconstrained, the liquid metal will continue to stretch until it reaches an instability point, whereupon the sudden release of surface energy causes an explosive break‐up of the droplet (Figure 1A top, Figure S1, Supporting Information). This behavior demonstrates the great potential of using Lorenz forces and fluidic instabilities to transduce significant electrical power into hydraulic power; here the dynamic breakup of a single droplet liberates 0.1348 J. By adding boundaries to the system, we establish a reactive pressure which acts to keep the liquid metal together upon breakup, reducing explosive breakup (Figure 1A middle). By further constraining the liquid metal droplet to a microchannel, we harness these fluidic instabilities and convert input energy into a more controlled and sustainable form of droplet separation, creating a stable repeatable cycle of liquid metal shapeshifting which can be used to generate fluidic flow and pressure (Figure 1A bottom).

Demonstrating the fundamental principles of liquid metal shapeshifting, we introduce a lightweight, deformable, embeddable, liquid metal valveless soft pump which utilizes low DC voltages – in contrast to most previous soft pumps^[^ ^18^, ^19^, ^20^, ^21^, ^22^ ^]^ – and operates on a fundamentally different mechanism from other liquid metal pumps.^[^ ^8^, ^10^ ^]^ The shapeshifting pump consists of a liquid metal droplet confined between two electrodes in a microfluidic channel with a small magnet placed under the droplet (Figure 1B,C). A DC voltage in the order of millivolts is applied to the electrodes, inducing current flow in the liquid metal. The moving charges within the liquid metal pass through the magnetic field and generate a Lorentz force.^[^ ^23^ ^]^ The Lorentz force opposes the surface tension of the liquid metal, causing the droplet to deform and elongate along the channel, generating pressure on the surrounding liquid. At a certain, repeatable stretch, instability particular to thin fluid films occurs^[^ ^24^ ^]^ and the droplet breaks into two, interrupting the current flow and thereby eliminating the Lorentz force. In the absence of the Lorenz force, surface tension causes the two separated parts of the droplet to rapidly return to their low energy conformation and facilitate droplet reintegration. Reintegration closes the electrical circuit and initiates a new cycle of Lorentz force‐induced deformation (Video S2, Supporting Information).

Characteristic instabilities, deformations and patterns are commonly observed at fluid‐fluid boundaries, typified by the formation of raindrops during rainfall.^[^ ^25^ ^]^ Likewise, liquid form a jet when it exits a nozzle, subsequently breaking into droplets due to changes in interfacial tension via the Rayleigh‐Plateau instability (Figure S9, Supporting Information).^[^ ^26^ ^]^ Post‐critical behavior, dependent on viscosity ratios, has also been shown in drop breakup^[^ ^27^, ^28^ ^]^ and in liquid bridges where, akin to LMSS, a droplet is stretched between two (typically spherical) anchors (Figures S8 and S9, Video S5, Supporting Information). ^[^ ^29^ ^]^ These instabilities have also been used to drive the breakup of immiscible fluids at T‐junctions in microfluidic channels.^[^ ^30^, ^31^ ^]^ In contrast to previous studies, here we exploit the coupling of instabilities with spatial asymmetries driven by the Lorentz force to generate an efficient emergent cycle of droplet breakup and coalescence, which facilitates the facile transduction of electrical energy to fluid flow.

## 2 Results

### 2.1 Principle of Operation

To analyze the emergent deformation cycle of LMSS we partition the cycle into four distinct stages (Figure 1D; Figure S17, Supporting Information); 1. Thrust: The droplet starts in a configuration with minimal surface area and shortest path between the anchoring electrodes. This permits the maximum current to flow through the liquid metal, generating Lorentz forces that push the liquid metal along the channel. Because it remains anchored to the electrodes, a curved neck is formed as the liquid metal is stretched. The fluid surrounding the droplet is pushed forward due to the generation of positive hydraulic pressure at the head and negative pressure at the tail of the deforming droplet. 2. Pinch‐off: As the liquid metal droplet is stretched, a neck is formed, the width of which decreases rapidly until the stability threshold is reached. During this phase, some of the surrounding liquid is pulled forward, encapsulated by the elongated liquid metal (see Figures S2 and S5, Supporting Information). 3. Rupture: When the surface energy of the droplet exceeds a critical value, the liquid metal droplet breaks into two smaller droplets, one anchored to each electrode. Upon rupture, the current flow and Lorentz force fall instantly to zero. Surface tension in the liquid metal now causes each of the two smaller droplets to retract into their respective minimum energy, lowest surface area, shapes. The fluid behind the droplet, which has moved forward with the shape change in stages 1 and 2, remains in front of the liquid metal due to the asymmetry in actuation (power stroke) and return paths (recovery stroke) of the liquid metal (see Figures S9 and S23, Supporting Information). 4. Coalescence: As the two smaller droplets return to their lowest energy shapes around their respective electrodes, their separation reduces to zero and they recombine to form one large droplet. As the droplet coalesces, it acts as a valve and prevents the fluid that has been pushed forwards from returning. Upon coalescing, the electrodes are once more connected, current flows, and the Lorentz force is reestablished. This four‐stage cycle is employed directly in the LMSS pump.

During the thrust and pinch‐off stages of the LMSS pump cycle, a cylindrical neck is formed. The liquid metal in this region is accelerated perpendicular to the current flow (which follows the increasing curvature of the deformed droplet), causing the necked area to thin until it ruptures due to thin fluid film instability.^[^ ^24^ ^]^ We have demonstrated that the droplet reaches instability in our system at an electric current of around three Amps. Below this threshold, the droplet reaches a stable elongated shape which does not approach instability and therefore does not break. We define the strain, or perimeter ratio, (ε) applied on the liquid metal by the Lorentz force as the ratio of the perimeter of a stretched droplet to its initial perimeter (before current is applied). This enables us to explore the maximum extension of a droplet before it ruptures (Figure S3, Supporting Information). Our results show that to reach instability (and hence initiate oscillation) the perimeter ratio must reach a minimum value of ε = 1.8 to overcome the surface tension of the liquid metal. Using a syringe pump, we explored droplet elongation without Lorentz forces and observed that the morphological alterations during rupture closely mirror those occurring at the aforementioned critical perimeter ratio (see Figure S13 and Video S6, Supporting Information.). In this context, the interfacial tension within the channel serves to counteract the Lorentz force, sustaining the droplet until it attains the critical perimeter ratio. The dimensionless Weber number (We) can define this critical dimension via the ratio of Lorentz forces to interfacial forces (see supporting text section 1.3). Our calculations show that at the critical perimeter ratio (Figure S3, Supporting Information), the forces are approximately equal (i.e., We ≈ 1) (see supporting text section 1.3 for calculations).

In the last stage of the LMSS pump cycle, droplets undergo coalescence, a crucial process that breaks the symmetry of the LMSS pump cycle (see Figures S5 and S9 and Video S5, Supporting Information). The coalescence of droplets involves three primary steps: 1) The two previously deformed anchored droplets are pulled back to their respective electrodes due to surface tension, where they collide and deform but do not yet fuse; 2) The continuous phase liquid film (NaOH) between the droplets is squeezed out; and 3) The interfacial film between the droplets ruptures, leading to fusion.^[^ ^32^ ^]^ As the film reaches a critical minimum thickness, Van der Waals and other inter‐atomic forces become dominant, initiating membrane rupture and droplet coalescence.^[^ ^33^ ^]^ Once the two droplets have combined, current starts to flow through the droplet and the LMSS pump transitions back to the power stroke.

By simply applying a low DC voltage to the LMSS pump, fluidic oscillator is created, and a hysteretic cycle is established with distinct power and recovery strokes, resulting in directional pumping (see Figures S9 and S10 and Video S5, Supporting Information) (Figure 1F). Oscillations and pulsatile flows are important in biology for the flow of body fluids, and the release of biochemicals is often cyclic or pulsatile.^[^ ^22^, ^34^ ^]^ The resulting oscillating pressure, demonstrated in Figure S10 (Supporting Information), can also be harnessed as a clock signal for a fluidic computational circuit.^[^ ^35^ ^]^ The capability to facilely generate pressure, flow and oscillations within a robot paves the way for constructing compact, entirely soft, untethered robots that can dynamically respond to environmental stimuli.

### 2.2 Characterizing the Liquid Metal Shapeshifting Pump

We investigated various parameters (as shown in Figure S2, Supporting Information) to optimize emergent shapes for maximum fluid pressure and flow rate. Our study examined the effect of electrode configuration, and we found that asymmetric electrodes led to more asymmetrical shape alteration and increases pumping performance. We also observed that lower values of current (which is proportional to Lorentz force) generated more symmetric shapes than higher currents. The forces due to surface energies for small fluid bodies, scale macroscopically with lengths, suggesting that a surface tension‐based actuator will be more effective at small scales.^[^ ^36^ ^]^ Therefore, as the height of the channel increases, the surface tension of liquid metal is not sufficient to return the droplet to stage 1 of the cycle, as the Lorentz force applied is much higher than the surface tension. We observed that in taller or open channels, small droplets of liquid metal are released into the flow (Figure 1A middle). ^[^ ^7^ ^]^ We also changed the channel width but did not observe a difference in the formation of the shapes.

**Figure 2:**
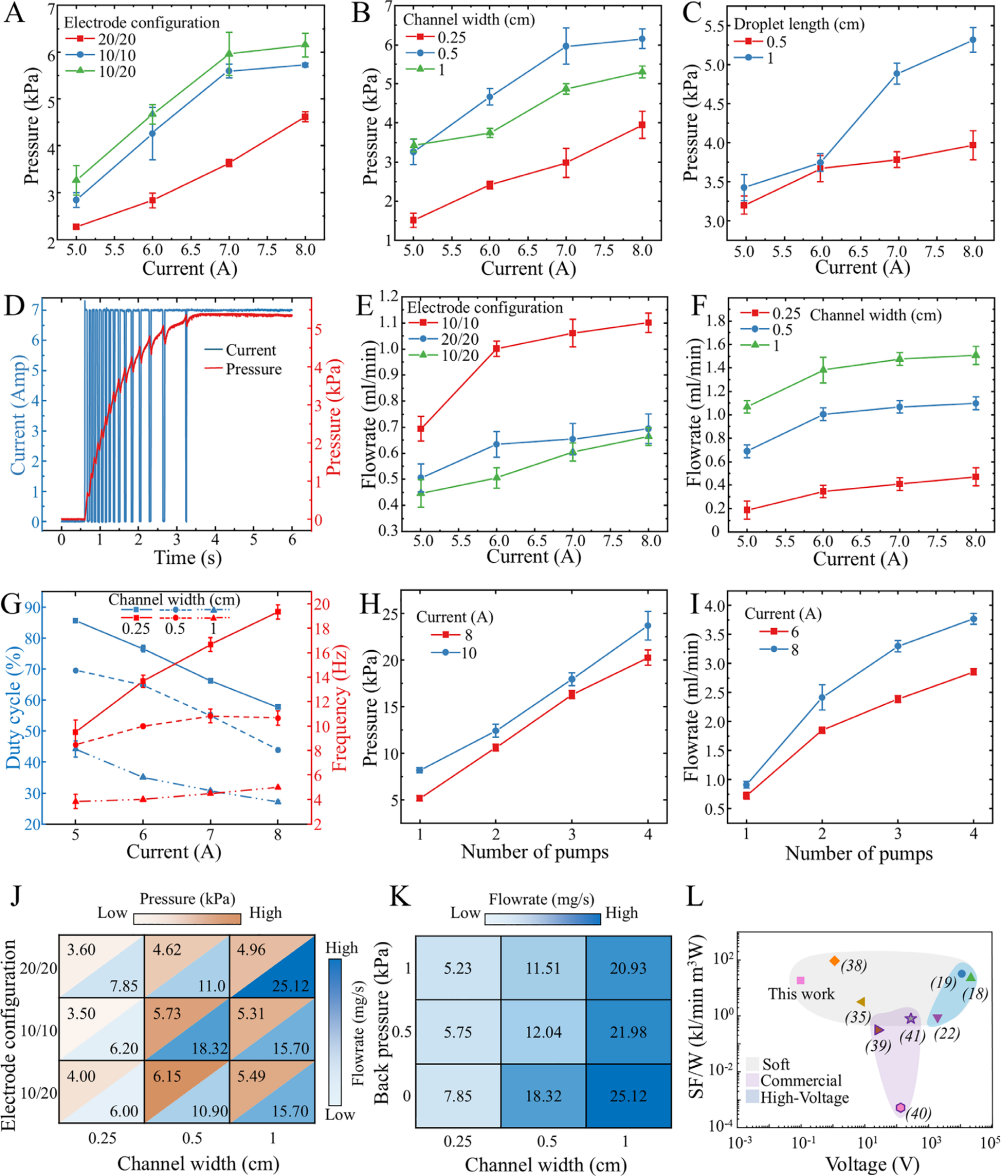
Performance characterization of the LMSS pump. A) Pressure versus current for different electrode configurations (10/10 is symmetric pins intruding inside the channel 10% of the width, 20/20 is symmetric pins intruding inside the channel 20% of the channel width, and 10/20 are asymmetrical electrodes at 10% and 20% insertion). Error bars represent the standard deviation of three measurements. B) Pressure versus current for different channel widths. C) Pressure versus current for different droplet length. D) Ramp‐up response of the pump against a closed output. The pressure increase at each cycle can be observed. E) Flowrate versus current for different electrode configurations. F) Flowrate versus current for different channel widths. G) Emergent frequency and duty cycle against current for different channel widths. H) Pressure from series connected LMSS pumps. I) Flowrate from parallel connected LMSS pumps. J) Pressure and flowrate for different channel widths and electrode configurations. K) Flowrate against back pressure for different channel widths. L) Comparison of specific flowrate per Watt versus operating voltage of LMSS pump and other soft and commercial pumps.^[^ ^38^, ^39^, ^40^, ^41^ ^]^ The detailed data are presented in Table S1 (Supporting Information).

Furthermore, our investigation explored the influence of electrical polarity on droplet asymmetry and determined that there is no discernible correlation. We demonstrate bidirectional pumping solely by reversing the direction of current (Figure S6 and Videos S3, S10, Supporting Information) and show high flow rates achieved by the LMSS pump using NaOH pumped fluids. Notably, the liquid metal droplet can function as a valve against high pressure during current flow, and can withstand back pressure (a single droplet can withstand 3 kPa) when inactive (see Figure S15, Supporting Information).

We developed deformable, embeddable pumps based on the LMSS pump concept, that are highly suitable for miniaturization and portable soft systems. Moreover, the ability to embed soft pumps within soft robots, such as artificial muscles, enhances the overall efficacy and durability of these systems.^[^ ^37^ ^]^ These pumps are silent, produce minimal vibrations and are compact (e.g., 15 mm long, 2 mm thick, and 7 mm wide, with fluidic channels measuring 15 mm long, 5 mm wide, and 0.1 mm high) and lightweight (0.45 g). We used PDMS as the pump body material, which has a low Young's modulus, high strain at rupture (>100%) and is widely used in microfluidics. We utilized established elastomer processing technologies, which are highly reproducible and can be readily scaled for industrial production. Our tests were performed primarily using NaOH as the pumped fluid, as it produced the best pumping performance. However, the system could also operate for a limited time with air and NaCl solution. In these media, liquid metal oxidation occurs which limits the stable operation of the pump.^[^ ^8^ ^]^ Nonetheless, this capability enables the pump to operate for short periods with liquids of neutral pH and in the presence of air bubbles (Figure S6 and Video S10, Supporting Information).

The LMSS pump operated at very low electrical voltages (the mean ranging from 0.01 to 0.1 V) and low power (ranging from 0.006 to 0.4 W), allowing it to be powered directly from a single AAA alkali battery. Utilizing a power supply with current and voltage limit control allows the pump to operate without electrolysis. Simpler power supplies without this option can use a basic voltage clamp or switch to prevent electrolysis (refer to Figure S32, Supporting Information). The internal resistance of alkali batteries also eliminate electrolysis. The fabrication cost of the LMSS pump prototype is less than 45 UK pence (57 US cents), which can be significantly reduced through mass production. The pump is capable of generating flowrates and pressures suitable for macroscale and microscale applications. Safe operation on or in the human body is possible since the electrical voltage is far below the human safety threshold.^[^ ^42^ ^]^ The intrinsic compliance, low mass, and low power consumption make the soft pump an enabling tool for microfluidics, portable soft robotics and fluid‐based wearable devices.

Table S1 (Supporting Information) showcases the essential performance metrics of the deformable LMSS pumps, encompassing specific pressure, specific flow rate, and power consumption, along with an overview of hydraulic pumps operating on other principles. Notably, a single LMSS pump exhibits a specific pressure of 33.33 GPa⋅m^−3^ and a specific flow rate of 7.71 kl⋅min^−1^⋅m^−3^, surpassing published micro‐pumps and commercial pumps, particularly in specific pressure, while operating at the lowest recorded voltage. The LMSS's capability for pumping can be customized by changing the applied current, electrode configuration, channel width, and magnet placement. The Methods explain experimental configurations for testing pumping performance. As illustrated in Figure S2 (Supporting Information), the morphology of the liquid metal during elongation can be asymmetrical or symmetrical and is influenced by factors such as the orientation and intensity of magnetic field lines, electrode arrangement, electrode insertion depth, and applied current. The force of the power stroke can be increased by increasing current or by increasing the strength of the magnet, up to a threshold where the anchoring of the liquid metal to the electrodes is exceeded.

The results of the characterization experiments are presented in **Figure** **2**. As shown in Figure 2A, the pressure generated increases with current and asymmetrical electrode configuration, with maximum pressure observed for channel width 0.5 cm (Figure 2B–D). Asymmetric electrodes exhibit superior anchoring effects (material and methods) and heightened stability against back pressure. Increasing current also generates higher flow rates, with the highest flow rate observed for a channel width of 10 mm and symmetric electrodes with an intrusion of 0.5 mm (Figure 2E,F). The emergent frequency and duty cycle (ratio between separated and coalescence stages) was measured for applied currents between 5 and 8 Amps. An increase in current corresponds to a rise in frequency, alongside a decrease in duty cycle (Figure 2G). This observation suggests that droplet breakup time is dependent on current and hence developed Lorentz force, while the coalescence time remains relatively unaffected, primarily governed by material and chemical properties.

Our investigation explored the arrangement of LMSS pumps in both series and parallel configurations, revealing a phenomenon wherein spatially separate droplets tend to self‐organize, resulting in elevated pressure and flow rates when deployed in such arrangements (Video S9, Supporting Information). We demonstrate the pump's modularity: One, two, three, or four LMSS pumps are fabricated in series to measure the total pressure (Figure 2H and S11, Supporting Information) and in parallel to measure the total flow rate (Figure 2I). The generated pressure scales with the number of series‐connected pumps and the flow rate scales with the number of parallel‐connected pumps, reaching a pressure of 24 kPa in the 4‐unit series configuration and a flowrate of 3.72 (ml⋅min^−1^) in the 4‐unit parallel configuration which enables the creation of high performance soft macroscale pumps. To evaluate the extent to which LMSS pumps can self‐organize, we define the synchronization metric as the ratio of the time the emergent cycles of the LMSS pumps are overlapping (on/off) to the total time. We achieved 71% synchronization in two LMSS pumps in series configuration. A simple model for the series and parallel configuration is demonstrated in Table S2 (Supporting Information).

From the Lorentz force equation, we can increase the induced force either by increasing the current or by increasing the magnetic field strength. We simulated stacked LMSS configurations with and without a closed magnetic circuit (Supporting Information section 1.6). COMSOL simulation results show that we can increase the magnetic field more than 8 times and decrease the power required 64 times to 0.006 W by closing the magnetic circuit. As shown in Figures S21 and S22 (Supporting Information), our specific pressure and specific flowrate per Watt reach 5332 GPa⋅m^−3^⋅W^−1^ and 1234 kl⋅min^−1^⋅m^−3^⋅W^−1^ surpassing previous soft hydraulic pumps and commercial pumps.

A design guide has been developed to streamline the customization of pump parameters to meet diverse performance criteria (Figure 2J–L). Based on the required flowrate and the pressure needed, the user can readily determine the suitable channel width and its electrode configuration. This resource facilitates the tailored design of pumps optimized for high flow rates or pressures, thereby addressing specific operational requirements. It can be seen, for example, that a channel width of 5 mm with an asymmetrical electrode configuration (10% – 20%) achieves the highest pressure, while a channel width of 10 mm with a symmetrical electrode configuration (20% – 20%) yields the highest flow rate, both with and without backpressure. Additional detailed plots can be found in Figure S14 (Supporting Information).

Given the deformable nature of the pump, we investigated its performance under deformed conditions. The back pressure caused by the reduction in channel height^[^ ^43^ ^]^ led to only a minor decrease in the performance of the pump (Figure S7, Video S11, Supporting Information). Additionally, we demonstrated the reliability and durability of the pump by operating it for over seven thousand cycles (Figure S12, Supporting Information).

### 2.3 Powering Fluidic Soft Robotics

**Figure 3:**
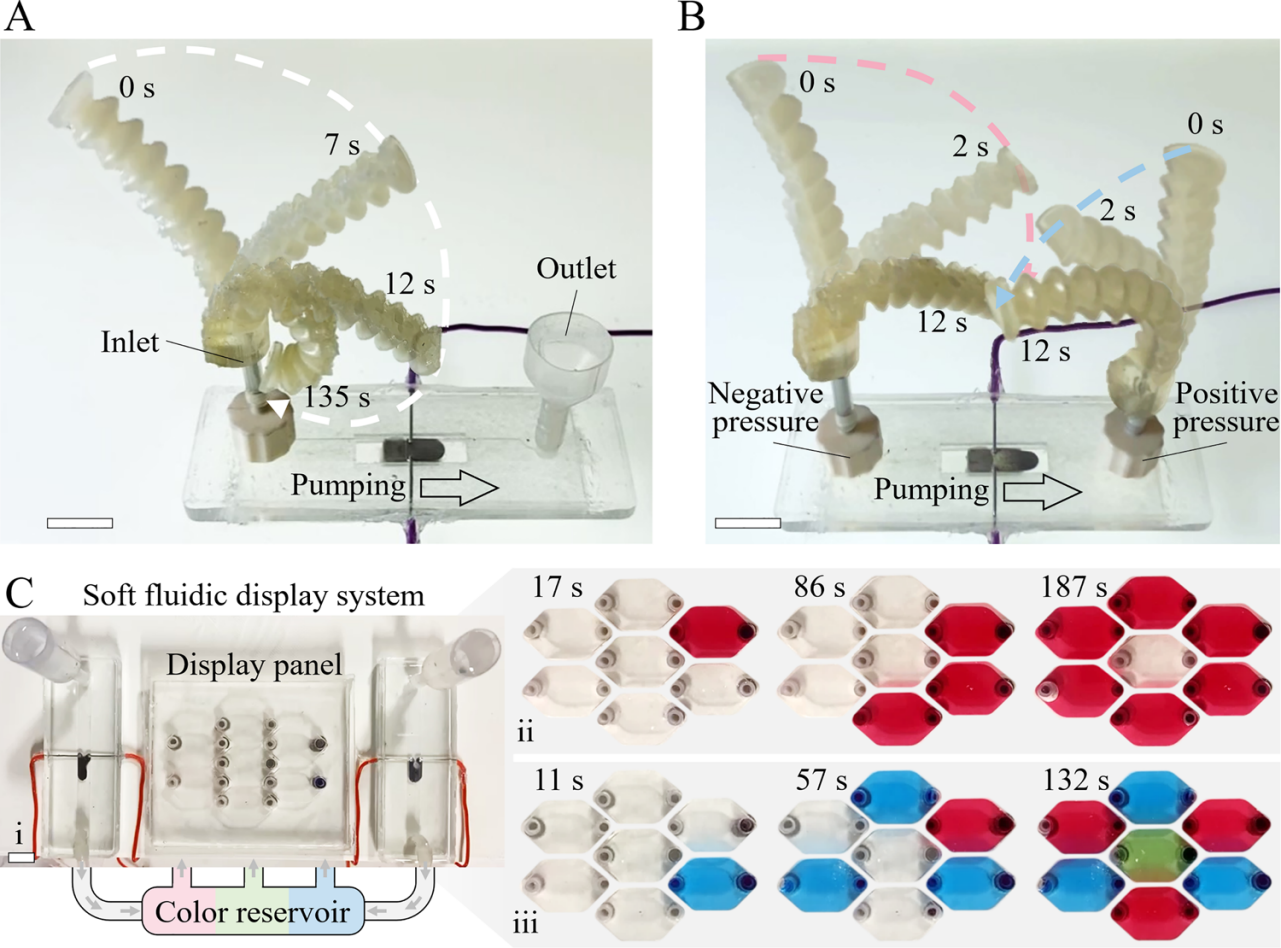
Demonstrating fluidic capabilities of the LMSS pump. A) A soft 3‐D printed fluidic actuator is powered using a single LMSS pump. The actuator bends when a voltage is applied to the pump: the soft pump moves the liquid (as well as air) from the actuator to the reservoir, causing it to curl up. B) By placing one soft actuator in the inlet and one at the pump's outlet, we can obtain antagonistic motion from two soft actuators due to the negative and positive pressure generated by the LMSS pump. Video S7 (Supporting Information) shows the fluidic actuator in action. C) Soft fluidic display units: i. Replicating the chromatophores of zebrafish, cells are filled with red, green and blue colored fluids by an LMSS pump, thereby functioning as small pixel units for wearable applications. ii. Employing one LMSS pump, we circulate red color fluid into the soft units to create a flower pattern. iii. Utilizing two LMSS pumps and a three‐way valve, we circulate three different colored fluids to illustrate a photo reflective RGB display. A detailed diagram of the connections is shown in Figure S27 (Supporting Information). Scale bars are 1 cm.

The ability of the LMSS pump to generate substantial flow rates and pressures while maintaining flexibility enables many applications in fluidic devices, soft robotics and wearable applications, which previously required a bulky external pump. We have demonstrated the LMSS pump in the context of fluidic actuators to demonstrate its high pressure capability, dynamic fluidic units capable of camouflage to show its high flowrate feature, and a wrist pump capable of driving a photoprotection skin to highlight its compactness and portability. **Figure** **3**A depicts a soft three‐dimensional printed fluidic actuator powered and controlled by the negative pressure generated by a single LMSS pump at its input. Upon applying electrical energy, the actuator reaches a deflection exceeding 90 degrees relative to its initial position in less than 12 seconds. In Figure 3B, we exploit the positive and negative pressures generated by the LMSS pump to drive two soft actuators in an antagonistic configuration simultaneously. We also studied the tension force generated and displacement achieved by a linear pouch motor driven by the LMSS pump. These actuators can form the basis for developing subsequent generations of soft robots, combining the durability, substantial deformation capacity, and adaptability of fluidic actuators with compact, efficient and embeddable LMSS pumps (Figures S25 and S26, Supporting Information).

Fluidic systems play a pivotal role in many processes, enabling heat, mass, information and energy transport. Here, we show the application of an LMSS pump to circulate red, green and blue fluid into soft pixel units, or chromatophores, which can be integrated into smart clothing (Figure 3C). Manipulating the color of these display units enables dynamic adaptations for camouflage, drawing inspiration from the natural color changing mechanisms in the skins of teleost fishes.^[^ ^44^ ^]^ We circulate red fluid into the pixel units (Figure 3Ci) using one LMSS pump to create a flower‐shaped pattern (Figure 3Cii). Using two LMSS pumps we circulate red, green and blue fluid to demonstrate the potential of our system to generate any RGB color and to act as a building block for smart clothing (Figure 3Ciii).

**Figure 4:**
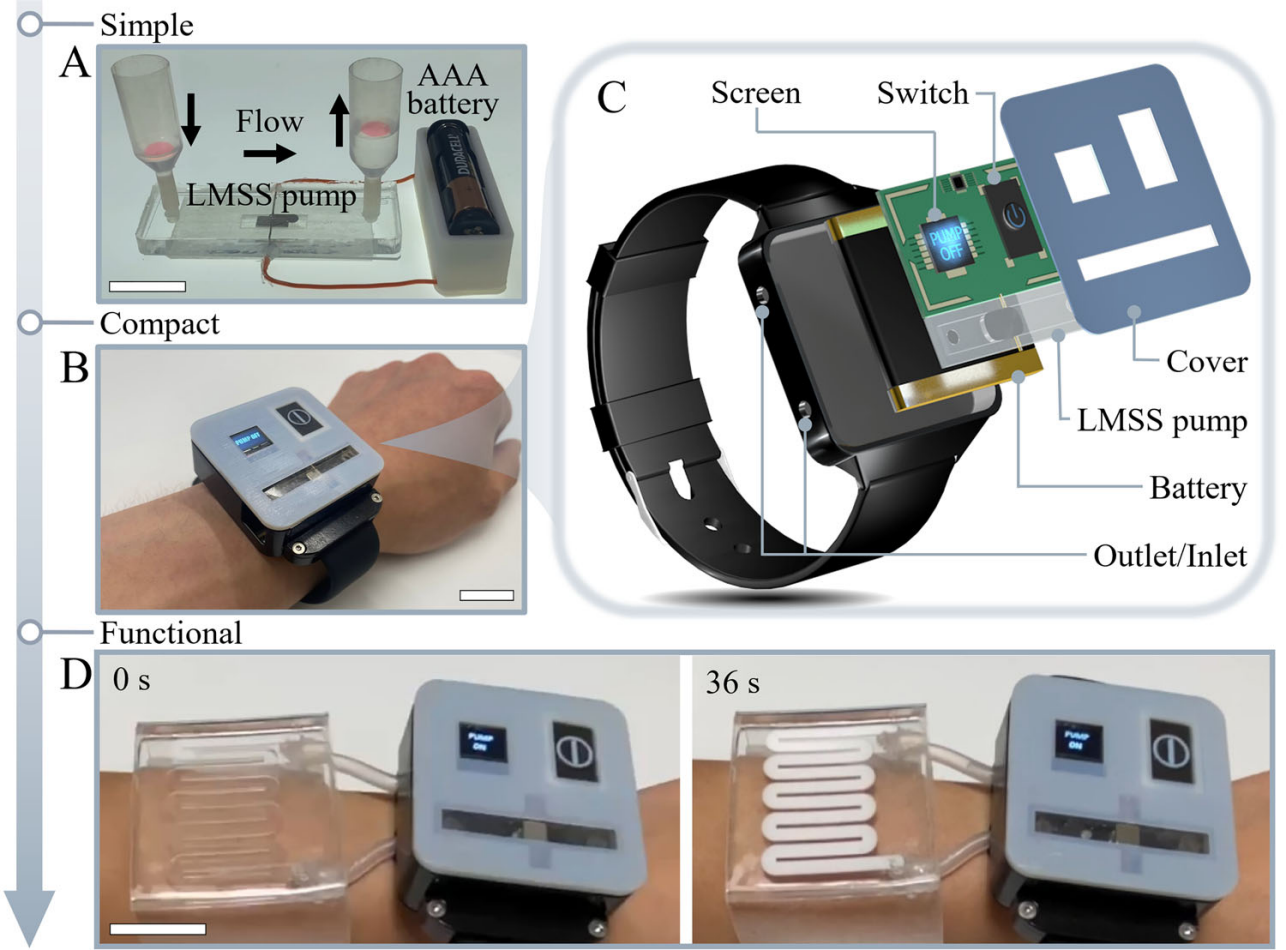
Demonstrating a compact, portable, battery‐powered wrist‐pump for dynamic skin‐protection. A) LMSS pump can be powered directly from AAA battery without any control circuitry. B) The wrist‐pump is compact enough to be worn as a daily accessory. C) Exploded view of the wrist‐pump containing the LMSS pump, LiPo battery to power the pump, a screen to show the state of the pump and a switch for control. D) The wristpump can dynamically protect the skin from harmful UV light by circulating a titanium dioxide suspension to block harmful radiation. Scale bars are 2 cm.

In **Figure** **4**A we demonstrate the simplicity of the LMSS pump by powering it directly from a AAA battery (measured during operation at 0.2–0.35 V, 6 Amps) without any control circuit for more than 8 minutes (Figure S24, Supporting Information). Moreover, we demonstrate the versatility and applicability of the LMSS pump and its compact design by incorporating it into a wristwatch capable of actively circulating fluid for various wearable applications (Figure 4B–D). The wrist‐pump comprises an LMSS pump, an indicator, and a small lithium‐polymer battery (Figure 4B,C). It serves as a versatile daily accessory for diverse wearable demonstrations. To ensure the pump's temperature is safe for wearable devices, we studied the temperature rise and demonstrated that it stabilizes at a safe temperature (Figure S16, Supporting Information). We developed a UV protection skin that can be activated by the LMSS pump (Figure 4D) in a dynamic mode (Figure S29, Supporting Information). The LMSS pumps an aqueous suspension of titanium dioxide through the skin, providing effective protection against UV light and capable of decreasing the UV‐A power by 40 percent to 0.6 (mW⋅cm^−2^) at a titanium dioxide concentration of 200 mg⋅ml^−1^. In Figure S28 (Supporting Information), we demonstrate the effectiveness of different concentrations of titanium dioxide in reducing the exposure rate to safe levels.^[^ ^45^ ^]^

## 3 Conclusion

We have introduced the liquid metal droplet shapeshifting principle, which enables the transduction of significant energy through the simple application of current to a liquid metal droplet in a magnetic field. By harnessing intrinsic instabilities in fluid‐fluid interactions, a cycle of Lorentz‐driven stretching and breakup, and surface tension‐driven recovery and coalescence, emerges autonomously.

This fundamental shapeshifting behavior has been embodied in the LMSS pump, a soft, compact, lightweight, low voltage, self‐oscillating liquid metal pump capable of generating high specific pressures and flow rates, surpassing previous soft and commercial pumps. The LMSS pump scales in parallel to generate high flowrates and in series to generate higher pressures; four‐unit pump can exert pressures of 24 kPa, with the largest possible pressure scaling of all soft pumps at 400 kPa m^−1^. Structural scaling predicts a 64 times reduction in input power and consequent increase in specific pressure per Watt (5332 Gpa·m^−3^·W^−1^) and flow per Watt (1234 kl·min^−1^·m^−3^·W^−1^), over ten times better than previous soft pumps.

This advancement removes the need for rigid and bulky external pumps from soft fluidic devices and simplifies electrical supply and control. The scalability, simplicity, stability of operation, low voltage and easy fabrication of the LMSS pump, including being directly driven from an AAA battery, is game changer in myriad embedded applications not possible with conventional technologies including soft wearables and smart clothing, medical devices, soft robots and micro/milli‐fluidic systems.

## 4 Experimental Section

### Manufacture of LMSS Pump

The LMSS soft pump consists of a flexible fluidic channel containing a solution of NaOH (1 M), a droplet of liquid metal (E‐GaIn), and two identical pin electrodes (ID 6mm) inserted into the microchannel from both sides and sealed with silicone glue (Sil‐poxy, Smooth‐On). The fluidic channel was constructed from two layers of PDMS, which were affixed through a plasma bonding process conducted using a Zepto plasma surface treatment machine (Diener Electronic, UK). One side of the PDMS layer features the microchannel, fabricated by mould casting in a 3D printed part using a J826 Prime 3D Printer. The lower PDMS layer contains a magnet (N52 high‐grade neodymium block in size of 5 mm × 5 mm × 1 mm, Guys Magnet, UK) embedded within the PDMS to align precisely beneath the electrode positions upon plasma bonding. The outlet apertures for the microchannel were established using a steel punch of 4 mm diameter.

### Characterization Experiments

In the pressure characterization of the LMSS pump, a pressure sensor (SSCDRRN060MDAA5, Honeywell, UK) was connected to the pump outlet. A raindrop sensor module (AZ, Deutschland) was employed to assess the flow rate. Data was recorded through a data acquisition system (USB‐6211, National Instruments).

This investigation explored pressure and flow characteristics under varying conditions, including different channel widths (ranging from 0.25 cm to 1 cm), pin insertion depths (ranging from 0.25 to 2 mm), and different lengths of liquid droplets (ranging from 0.25 cm to 1 cm). To keep the magnetic field uniform for these characterization experiments, a bigger magnet (50 mm × 25 mm × 10 mm) was used for these experiments at a height at which the field was very close to the field of the small magnet (5 mm × 5 mm × 1 mm). The details have been presented in Figure S19 (Supporting Information). After determining which of these parameters gave the best results, the LMSS pump was characterized using the small magnet for flow rate and pressure. The data was shown in Figure S4 (Supporting Information). Data points represent the mean value taken from three measurements for all experiments, and the error bars represent their standard deviation.

For all characterization experiments and demonstrations, the microchannel was first filled with NaOH (1 M). Subsequently, a droplet of liquid metal was injected into the channel to connect the two electrodes. The NaOH removes oxidation from the surface of the liquid metal by chemical reduction and increases its surface tension. For stable operation, the droplet must wet to electrodes, which forms a constraint for the droplet. Liquid metal (E‐GaIn) droplets anchor to metals such as copper due to metallic bonding.^[^ ^46^ ^]^ Copper electrodes (ID 0.6mm) were utilized and noticed that a quicker bonding effect was obtained by dipping the electrodes in liquid metal before insertion into this channel. Tinned copper electrodes (ID 0.6mm) were also studied and did not observe a difference in the bonding effect from copper or the pumping operation. As the droplets anchor to the electrode after contact, electrical contact was maintained, and a small restoring force was generated to the equilibrium position when the droplet was symmetrical about the electrodes.

To investigate the temperature increase in this system due to Joule heating, a thermal camera (FLIR E4) was used to monitor the pump's temperature at the most commonly used and the highest currents (6 Amps and 8 Amps) (Figure S16, Supporting Information).

### Soft Robotic Actuator Demonstration

The bending fluidic actuator was designed and crafted using SolidWorks software and 3D printed (J826 prime 3D Printer, Stratasys) using Vero black and Agilus clear materials. Upon connecting this actuator to the LMSS pump and delivering a current (8 Amps), the actuator bends due to the positive (outlet) or negative (inlet) pressure created by the pump. The bending was recorded with a camera, and the angle was computed using the digital image processing tools of MATLAB.

Moreover, a linear pouch motor actuator was fabricated by heat sealing (Impulse hand sealer, RS PRO, UK) flat tubes (LFT2120STK Polythene Layflat Tubing, Polybags, UK) with 0.1 mm thickness into a single pouch with internal dimensions 2.6 cm by 1.4 cm. The tension force generated using the linear actuator was measured with a 10 N load cell (SparkFun Electronics, UK), and displacement was recorded with a camera (RX10 IV, Sony) and computed using MATLAB's digital image processing tools (Figures S25 and S26, Supporting Information).

### Soft Fluidic Display Units

Soft fluidic display units, or artificial chromatophores, were constructed from two layers of PDMS, mould casted in a 3D printed mould, which was affixed through a plasma bonding process. One side of the PDMS layer features hexagon‐shaped chambers with a depth of 2 mm. The outlet apertures for the fluidic channel were established using a steel punch of 4 mm diameter. The chambers were connected to the LMSS pump(s) based on the desired display pattern. For single‐colored patterns, one LMSS pump was used, and for the three‐colored patterns, two LMSS pumps were used with a three‐way connector, which enabled them to switch the color of the pumped fluid for the desired chamber.

### Wrist‐Pump

The wrist‐pump comprises a miniature LMSS pump, a screen (ESP32 S3 development board), a switch, a PCB board and a small lithium‐polymer battery (3.7 V, 220 mAh) with dimensions of 25 mm × 20 mm × 7.5 mm. The wrist‐pump design was crafted using SolidWorks software, and 3D printed (J826 prime 3D Printer, Stratasys). The wrist‐pump was capable of powering and controlling different wearable soft robots. Using the wrist‐pump, controlling a UV protection skin was demonstrated, which was manufactured by mould casting a soft silicone rubber (Smooth‐On Ecoflex 0030) in a 3D printed mould. The wrist pump circulates a white fluid, a mixture of titanium dioxide (Special ingredients, UK) and water, into the UV protection skin. By employing a UV sensor (GUVA‐S12SD, DFRobot), the UV radiation was measured that can pass through the different concentrations of titanium dioxide aqueous dispersion.

### Series and Parallel

To test the LMSS pump in series, a 7.5 cm sample was created that included four sets of electrodes, 1.8 cm apart. This configuration will increase the pressure of the system. Additionally, pressure and flowrate characterization of LMSS pumps were conducted by connecting four typical samples in a series and parallel configuration to enhance the pressure and flow rate.

### Finite Element Analysis

The electric potential (V), current density (A⋅m^−2^) and the electric volumetric loss density (W⋅m^−3^) were simulated using the software COMSOL Multiphysics 5.6. The liquid metal droplet was modeled with a thickness of 0.1 mm, and its relative permittivity was specified as 1.

Supporting Information is available from the Wiley Online Library or from the author.^[^ ^47^, ^48^, ^49^, ^50^ ^]^

## Supporting information



Supporting Information

Supplementary Video 1

Supplementary Video 2

Supplementary Video 3

Supplementary Video 4

Supplementary Video 5

Supplementary Video 6

Supplementary Video 7

Supplementary Video 8

Supplementary Video 9

Supplementary Video 10

Supplementary Video 11

Supplementary Video 12

## Data Availability

The data that support the findings of this study are openly available in the University of Bristol Research Data Repository at https://doi.org/10.5523/bris.uroi0t11a4a52d1pt8x9uqjc8.
