# Supplementary material for: Shapeshifting Liquid Metal Droplets for Soft Fluidic Machines
Source: Adv Mater. 2025 Oct 23;38(11):2420265. doi: 10.1002/adma.202420265 (PMC12921347; doi:10.1002/adma.202420265)
Supplement: Supplementary file 1 — Supporting Information [file ADMA-38-2420265-s011.pdf]

Supporting Information

Shapeshifting liquid metal droplets for soft fluidic machines

*Saba Firouznia, Christian Romero, Hemma Philamore, Andrew Conn, Martin Garrad, Jonathan Rossiter**

* Correspondence to: jonathan.rossiter@bristol.ac.uk

Contents **Page**

1. Supplementary Text 2
2. Supplementary Figures and Tables 5
3. Supplementary Videos 45

1. Supplementary Text:

1.1 Liquid Metal Shapeshifting (LMSS) principle

LMSS operates through two principles:

1.1.1 Magnetohydrodynamics

Magnetohydrodynamics (MHD) is the study of how an electromagnetic field interacts with a conducting liquid, gas, or plasma. The subject can be divided into different branches based on the specific working medium utilised. Each branch has its own unique set of scientific and technical problems due to differences in the physical characteristics of the media being studied. Liquid-metal MHD is distinct from other branches due to its simple equations, methodology similar to conventional hydrodynamics of incompressible media, and comparatively simple experiments.

According to Faraday's law, the total Lorentz force applied to the liquid metal is given by Equation S1. Assuming a uniform distribution of current along the y-axis (between the electrodes),

$$|F| = \int jBd^3 = \frac{I}{lh} Blhw = IBw \quad \text{(S1)}$$

Where B represents the Magnetic field, j is the magnitude of the current density, I is the electric current through the liquid metal, h is the height of the channel, l is the length, and w is the width.

1.1.2 Surface tension

The surface tension of liquid metal, a fundamental property stemming from intermolecular interactions at the liquid's interface, plays a pivotal role in shaping its behaviour and morphology. Unlike conventional liquids, liquid metals exhibit unique surface tension characteristics owing to their metallic bonding nature, which is typically stronger and more cohesive than that of other liquids [44]. This phenomenon results in pronounced surface tension forces; hence, the droplets will decrease the interface area as much as possible to reduce the surface energy in the presence of surface tension [45].

Moreover, the high surface tension of liquid metals enables them to exhibit remarkable wetting properties, allowing for their facile manipulation and patterning on various substrates.

Understanding and harnessing the surface tension force of liquid metals is important in diverse applications ranging from electronics and energy systems to biomedical devices, where precise control over their droplet shape, wetting behaviour, and interfacial interactions is paramount.

Previous studies have shown that liquid metal wire can be lifted, rotated, and suspended within a solution, exhibiting various patterns due to the interplay of Lenz's law and Lorentz forces [17]. In contrast to earlier liquid metal shape manipulation methods [14,16], the Liquid Metal Shape Shifter (LMSS) exploits the multi-physics interaction of dynamic Lorentz forces – created within the liquid metal, which serves as a mobile current carrier – and the surface tension of the liquid metal itself. This interaction not only governs the breakup and coalescence of the liquid metal but also produces a novel auto-commutating fluid motor effect.

1.2 Instabilities

To reap the benefits of the presented LMSS emergent cyclic fluidic motion, it is important to understand the underlying physics that govern liquid metal droplet stretching and the conditions that lead to breakage. Liquids typically form droplets when they exit a nozzle, for example, when a water tap is turned on with a low flow rate. If the flow rate is increased, a jet – a cylindrical stream of fluid – is formed. However, interfacial tension rapidly drives jet breakup into droplets via the Rayleigh-Plateau instability [26].

It is known that this instability arises when the liquid column (or jet) length λ exceeds the column diameter 2R by a factor of about 3.13 (with a maximum strain ratio $\varepsilon = \lambda/(2R)$ approximately equal to π)

Such jets are found both in mundane streams, such as water flowing from a faucet, as well as in important applications, such as industrial sprays, fuel injection systems, the drawing of polymeric fibres, and inkjet nozzles [46]. This liquid behaviour derives from the existence of small perturbations in any physical system. All real-world flows have some non-negligible external disturbance that will increase exponentially in unstable systems. In general, this deformation of the column, called varicose perturbations, is represented as a series of periodic displacement sinusoids [26]. From the Young-Laplace equation, we can say the pinched section has higher pressure, and the bulging section has lower pressure, thereby producing an internal fluid flow due to the pressure gradient. This internal flux causes the growth of displacement amplitude, which externally initiates droplet deformation. The droplets form when the pinched areas rupture, and the bulged areas transform into spherical droplets.

A more suitable model of the LMSS system is a liquid bridge between two anchoring spheres, the separation of which can be increased [29]. Upon stretching, the liquid bridge (length λ) between two spheres (radius R) thins and forms a neck, becoming unstable at a maximum strain ε. The upper bound on the maximum strain ($\varepsilon = \lambda/(2R) \approx 0.81R^{-1/3}$) can be empirically calculated. In these systems, a stable liquid geometry becomes unstable due to the presence of external disturbances. This occurs at the point when the fluid system can only reach its minimal energy state by minimising its surface area through formation of droplets.

1.3 Weber number

In our system, the interfacial tension within the channel serves to counteract the Lorentz force, sustaining the droplet until it attains a critical perimeter ratio. The dimensionless number Weber number (We) can define this critical dimension via the ratio of Lorentz forces to interfacial forces. Our calculation shows that at the critical perimeter ratio, We ≈ 1 (forces approximately equal).

$$We = \frac{\text{Intertial forces}}{\text{Interfacial forces}} = \frac{Lorentz\ force}{Surface\ tension\ force} = \frac{IBw}{\gamma P} \quad \text{(S2)}$$

I is the current going through the liquid metal, B is the magnetic field, and w and P are the length and perimeter of the droplet, respectively, which we find by image processing of the droplet at its initial state.

The phenomenological parameter γ (N/m) is, in the most general sense, a measure of interfacial force per unit length of the interface, but its physical origin is system-specific. For a liquid, this parameter is the surface tension. To find γ, we use the Young's Laplace Equation S3. In this equation, w and h are the width and height respectively, θ is the contact angle between the liquid and the walls of the channel, and P is the pressure exerted at the inlet to insert the EGaIn into the microchannel.

$$P = 2\gamma \cos(\theta)\left(\frac{1}{w} + \frac{1}{h}\right) \quad \text{(S3)}$$

We measure the pressure required to insert the EGaIn inside the microchannel (0.1 mm height and 5 mm wide) filled with 1M NaOH to be ~3.5 kPa. We measured the angle between the EGaIn and the side wall of the channel to be ~220°, based on the top-down image of the EGaIn in the channel as shown in supplementary Figure S30.

1.4 PDMS microchannel characterisation

The dimensions of the microchannels in the PDMS components were measured using a stylus profilometer (Dektak XT, Bruker Ltd, UK). The measurements were recorded by positioning the samples with the microchannel oriented upward and by scanning a stylus probe (2 μm radius tip) over the channel, as depicted in Figure S18A. The resulting profile data is shown in Figure S18B and Table S3. The standard deviation from the data obtained is smaller than 3.4 μm, as shown in Table S3.

1.5 Measurements and simulations of the magnetic fields of the MHD magnets

Two rectangular neodymium magnets (N52 grade) were employed as static magnetic field sources. The larger magnet (50 mm × 25 mm × 10 mm) was utilised for the characterisation of the MHD micropump, while the smaller magnet (5 mm × 5 mm × 1 mm) served as the magnetic field source for the demonstrators.

Magnetic field intensities were mapped across two planes, at heights 160 μm and 8 mm above the small and big magnets, respectively. These heights replicate the heights of the liquid metal above the magnets in the characterisation setups (Figure 2, Figure S4) and demonstrations (Figure 3,4).

Magnetic field measurements were performed using a precision gaussmeter (GM08 gaussmeter, Hirst Magnetic Instruments Ltd, UK), recording the field strength at the respective distances from the magnet surfaces. The results revealed that the magnetic field intensity measured at 160 μm from the small magnet was comparable to that recorded at 8 mm from the large magnet, as demonstrated in Figures S19 and S20.

To further validate the experimental data, finite element analysis (FEA) simulations were conducted using Finite Element Method Magnetics (FEMM) software. The simulation results corroborated the gaussmeter measurements, confirming that the magnetic field of the small magnet at 160 μm was comparable to that of the large magnet at 8 mm (Figure S20).

1.6 Scaling study

Scalable soft pumps enable precise control over fluid flow across different scales, enhancing their adaptability and performance in diverse settings. This scalability allows for cost-effective customisation, integration with various platforms, and futureproofing of

technologies, facilitating innovation and market expansion. Moreover, the ability to tailor pump sizes to specific application needs ensures that a single pump design can be efficiently utilised in both small-scale laboratory experiments and large-scale industrial processes, thereby broadening the scope and impact of these soft pump technologies.

As mentioned before, LMSS pumps can be used in series and parallel configurations to increase pressure and flow rate. In this section, we introduce stacking configurations to take advantage of magnetic field sharing between channels. Moreover, to improve the magnetic field density, we can use thin, soft iron incasements as magnetic keepers, which are bars made from soft iron or steel placed across the poles of a permanent magnet to help preserve the magnet's strength by completing the magnetic circuit. Magnetic keepers also help to protect other metals from being attracted towards these poles by closing the magnetic circuit and help isolate the pump from external magnetic fields.

We undertook COMSOL modelling of the magnetic field for different stacked configurations. Results show (Figure S21, S22) that by adding a thin iron incasement, we can increase the field around 8 times. This enables us to decrease the current 8 times while achieving the same Lorentz force on the LMSS pump's liquid metal droplet. Therefore, the input power required by our system can decrease 64 times, resulting in a power of 0.006 W and an efficiency of ≈1.5 per cent.

2. Supplementary Figures and Tables

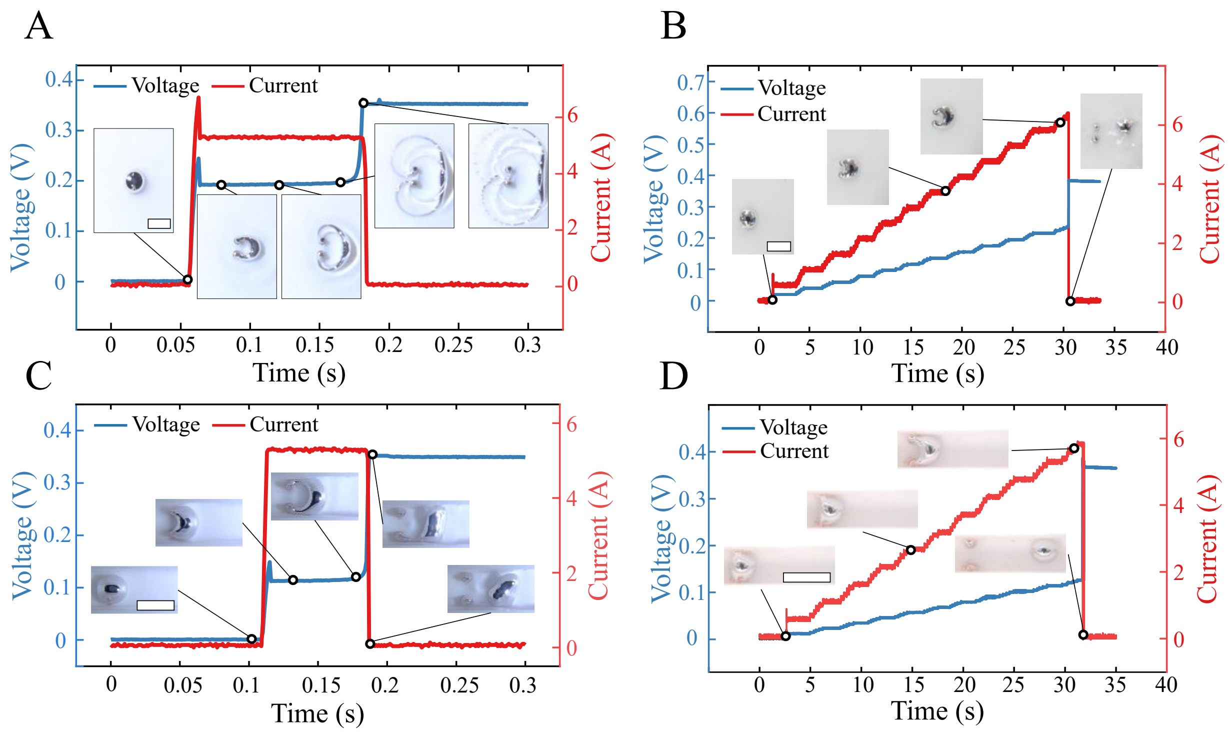

Figure S1. Droplet deformations in unconstrained and semi-constrained conditions. A-B) Voltage and current applied in a dynamic and quasi-static load to an unconstrained droplet. As shown, the droplet demonstrates huge deformation, which causes the droplet to explode. C-D) Voltage and current applied in a dynamic and quasi-static load to a semi-constrained droplet with side walls. The droplet shows more controlled shape changes and separates from the electrodes. Scale bars are 10 mm.

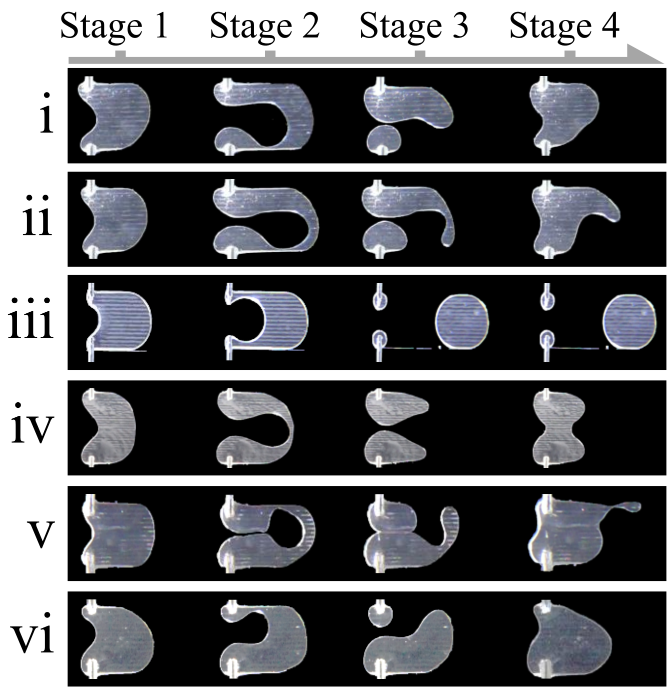

Figure S2. Effect of different parameters on liquid metal shape during the 4 stages of the pumping cycle: i. reference (0.5mm wide and 0.01 mm high channel, 8 Amps, magnet in the centre, symmetrical 0.5 mm electrode insertion depth); ii. effect of low current (3 Amps), iii. effect of channel height increase (0.5 mm); iv. effect of channel width increase (1 cm wide); v. effect of magnetic placement (LMSS pump placed on the lower end of the magnet); vi. asymmetric electrode insertion (0.5 mm and 1 mm). Except for case iii, all the other deformations cause pumping.

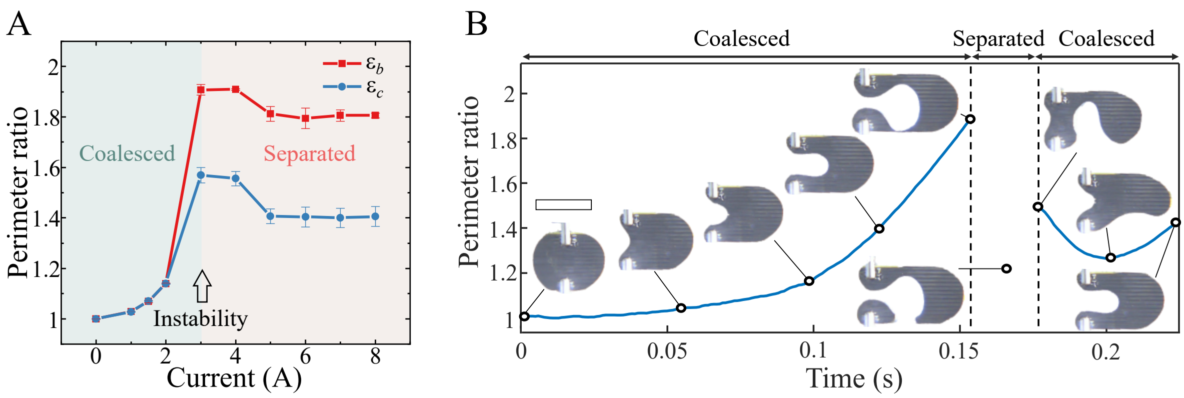

Figure S3. Perimeter ratio of liquid metal shapeshifting. A) Perimeter ratios for different currents at the point of rupture (ε_b, max perimeter) and after the coalesced stage (ε_c), demonstrating the instability and hysteresis. B) Perimeter ratio over one shapeshifting cycle. Scale bar is 5 mm.

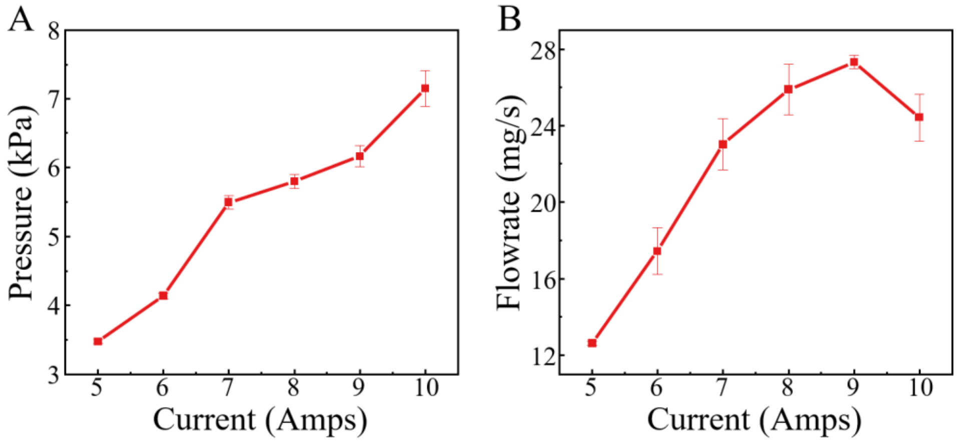

Figure S4. Small magnet LMSS pump characterisation. A) The pressure generated using different currents. B) The flowrate generated using different currents.

Stage 1

Stage 2

Stage 3

Stage 4

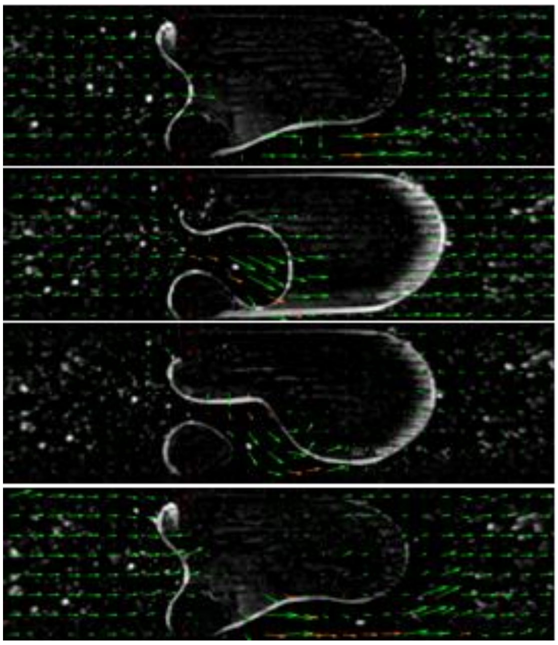

Figure S5. The particle image velocimetry (PIV) of LMSS pump. The trajectory of particles through the microchannel and the liquid metal droplet using a high-framerate camera (1000 fps) is demonstrated using PIV methods. 4 frames of this study are shown, depicting the 4 main stages (1-deforming, 2-pinch-off, 3-rupture, 4-coalescence) of the droplet in an LMSS pump.

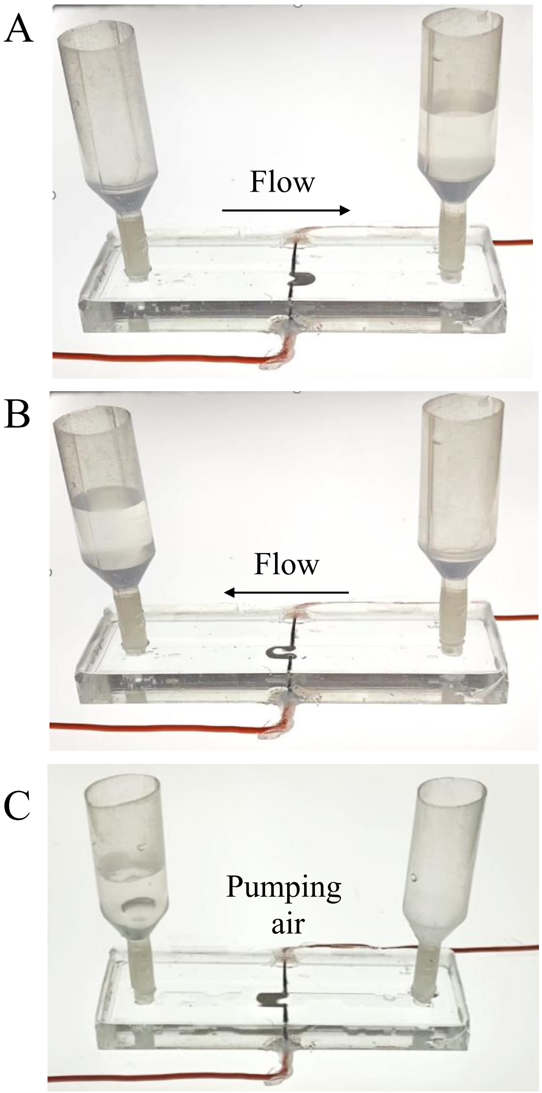

Figure S6. Demonstrating the bidirectionality of the LMSS pump as well as its capability to pump air. A-B) Demonstration of the LMSS pump moving NaOH between two reservoirs. By changing the polarity of the applied voltage, the flow direction can be reversed. C) Bubbles in the left reservoir demonstrate the LMSS pump's capability to pump air for a limited time. While air cannot substitute for the primary pumped medium (NaOH solution in our study), this observation confirms the pump’s effective operation, even in situations where air is trapped in the system.

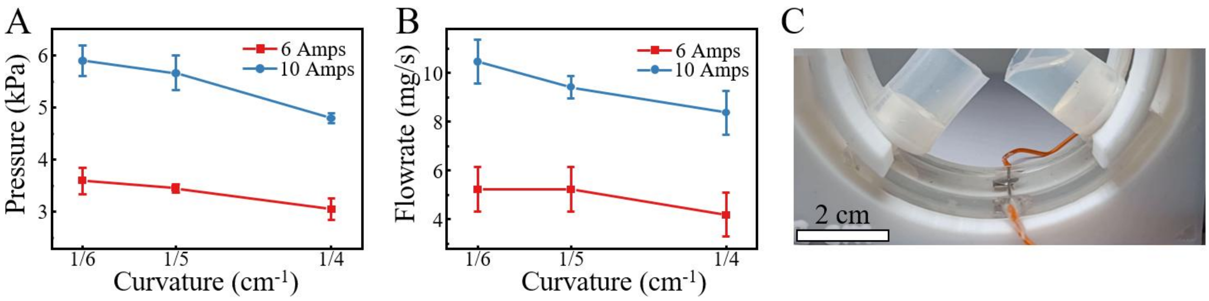

Figure S7. Performance of the LMSS pump at different bending curvatures. A) Pressure generated against different curvatures. B) Flowrate induced against different curvatures. B) LMSS pump deformed at the curvature of 0.25 cm^{-1}.

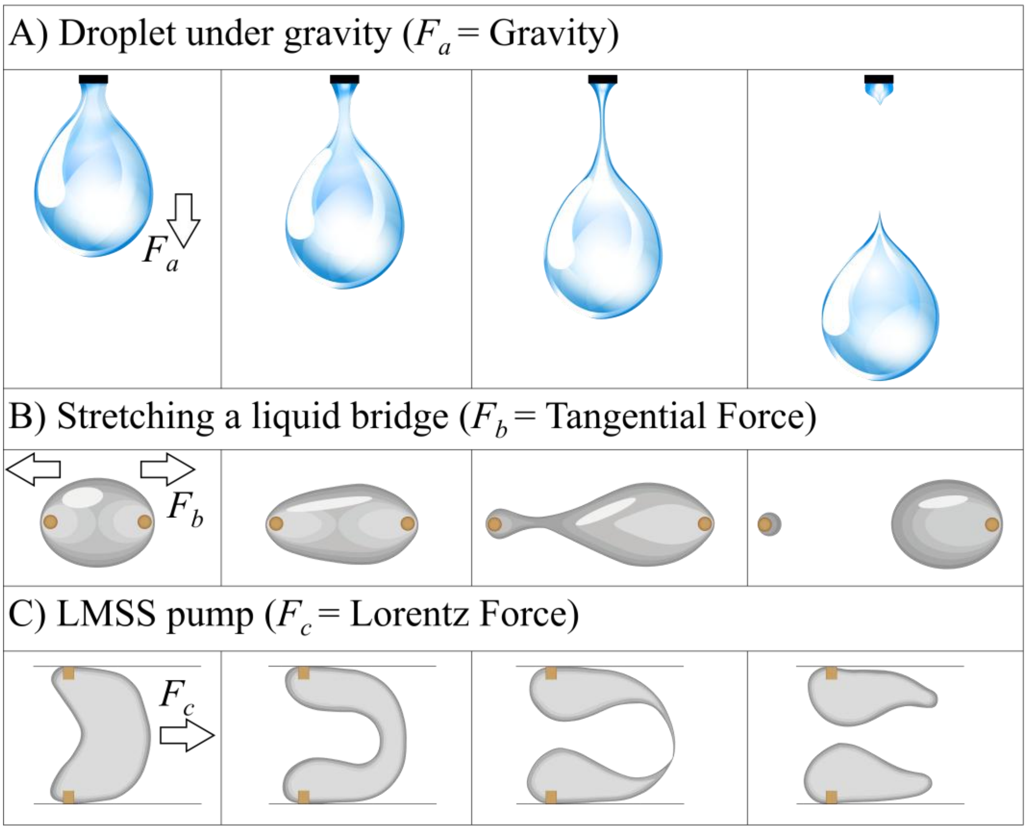

Figure S8. Diagrams of different stable liquid geometries becoming unstable due to the presence of external disturbances. These are driven by the same underlying mechanism; the fluid system reaches its minimal energy state by minimizing its surface area through formation of droplets.

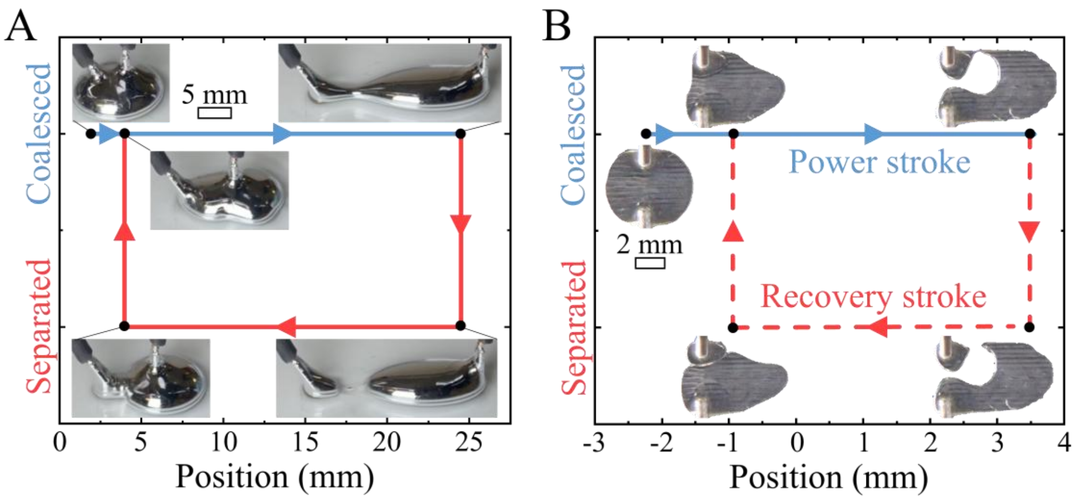

Figure S9. The hysteresis of liquid metal during mechanical stretching and actuation. A) Illustrating hysteresis in a basic system by employing two rods to confine a droplet of liquid metal. By fixing one rod while moving the other, we induce stretching and subsequent rupture. Reuniting the rods afterwards completes the cycle. B) We demonstrate the LMSS pump's hysteresis cycle, wherein the symmetry between the forward and backwards strokes is disrupted, resulting in an active and emergent cycle driving fluid propulsion. The droplet undergoes movement (power stroke) and stretching under the influence of the applied Lorentz force, leading to eventual instability and rupture. Subsequently, the recovery stroke is driven by the surface tension of the liquid metal, returning the cycle to its coalesced state. Throughout these cycles, we monitor the position of the rear of the droplet.

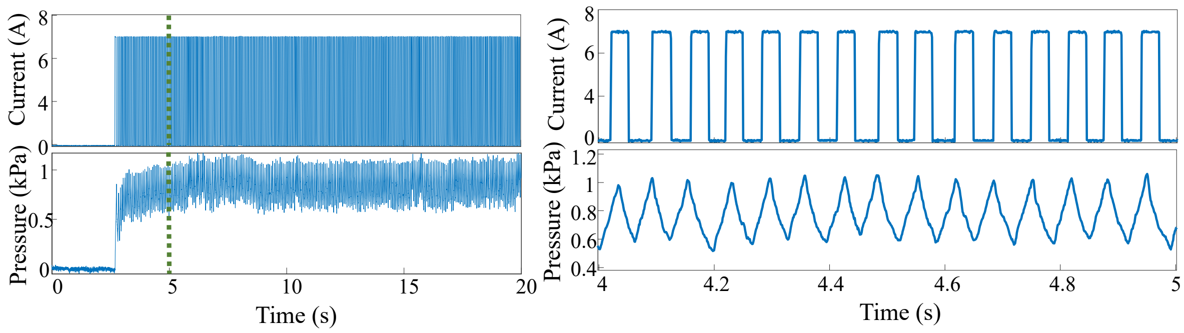

Figure S10. Active fluidic oscillator. Demonstrating the capability of LMSS pump as an active fluidic oscillator, we observe its capacity to generate current and pressure oscillations solely by connection to a direct current (DC) source. The pressure difference and frequency can be tuned by the current applied. The figure on the right is a magnified view of the area outlined by the dotted line in the left figure.

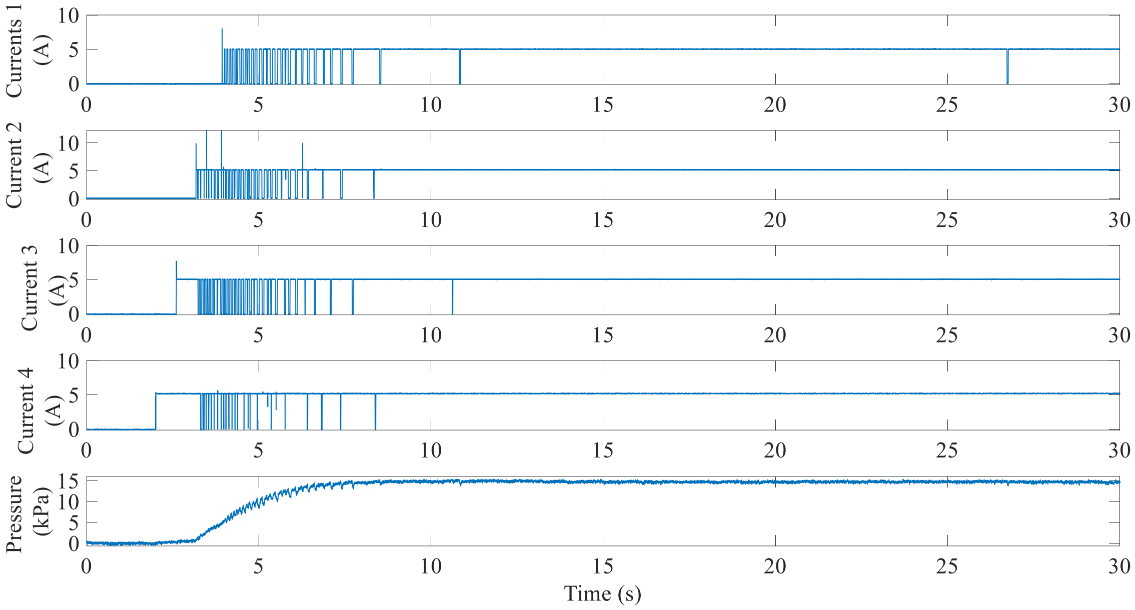

Figure S11. LMSS pumps in series. To increase the pressure of the LMSS pump, we can configure them in series. The data regarding the 4 droplets in series and parallel have been demonstrated in Figure 2.

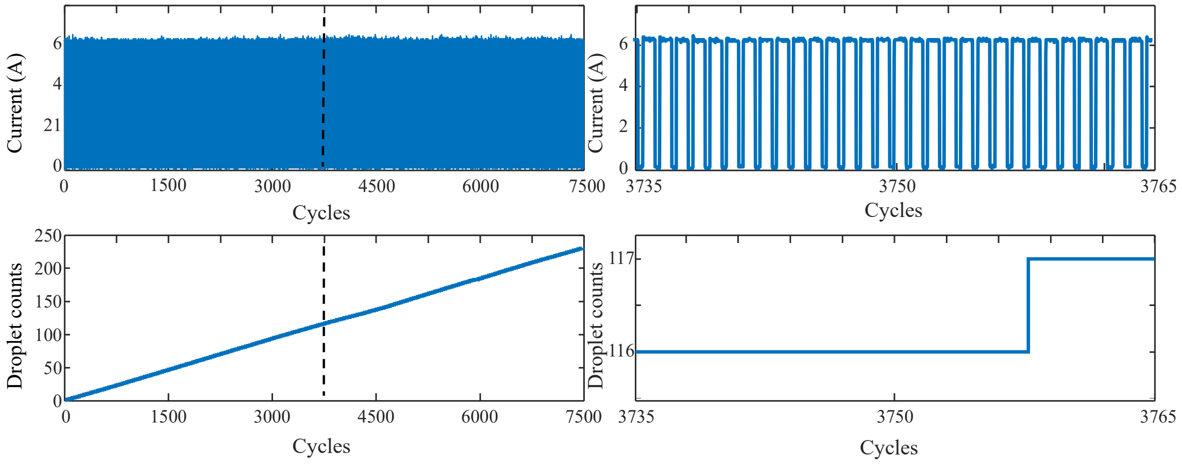

Figure S12. The reliability of the LMSS pump for over 7000 cycles. We show the applied current and track the pumped droplets using a droplet sensor. The figure on the right is a magnified view of the area outlined by the dotted line in the left figure.

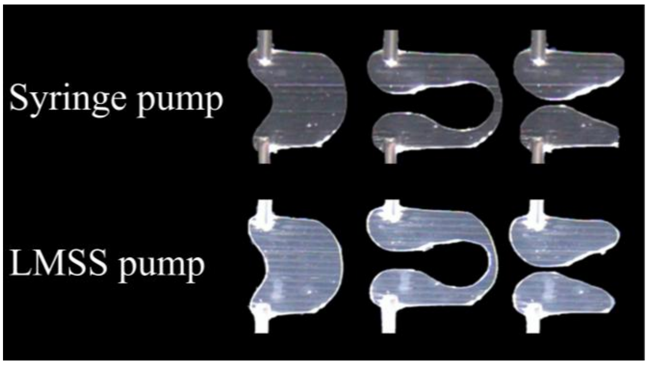

Figure S13. Comparing the emerged shape changes using the Lorentz force and syringe pump. We compared the shape changes generated by a syringe pump (flowrate of 0.1 ml/min) with the shapes generated by Lorentz forces (for a sample with a channel width of 5 mm and applied current of 3 A). It was observed that the emergent shapes were very similar, suggesting that droplet deformation is dominated by surface tension effects and that the Lorentz force acts independently of these.

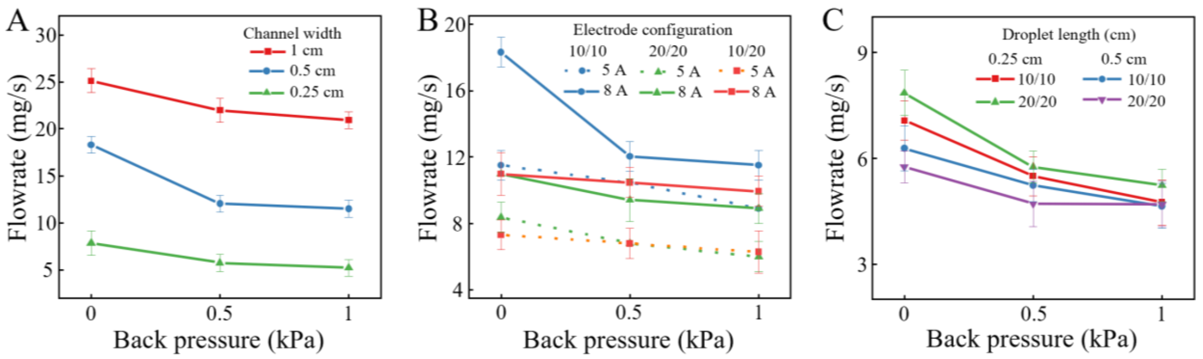

Figure S14. The detailed flowrate against different backpressures. A) Flowrate against backpressure for different channel widths. B) Flowrate against backpressure for different electrode configurations. C) Flowrate against different backpressures for different droplet lengths.

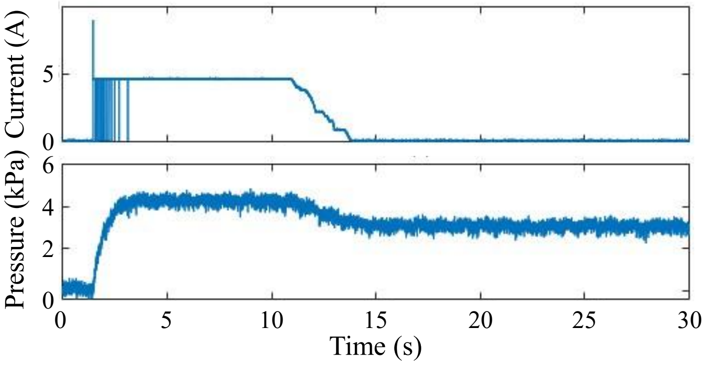

Figure S15. Demonstrating the valve capability of the LMSS pump. Sample data of pressure characterisation of the LMSS pump. We can observe that the droplet can hold pressure and act as a valve against a built up of pressure (~4 – 10 s). Moreover, we demonstrate that the LMSS pump is capable of holding pressure without current (>~15 s).

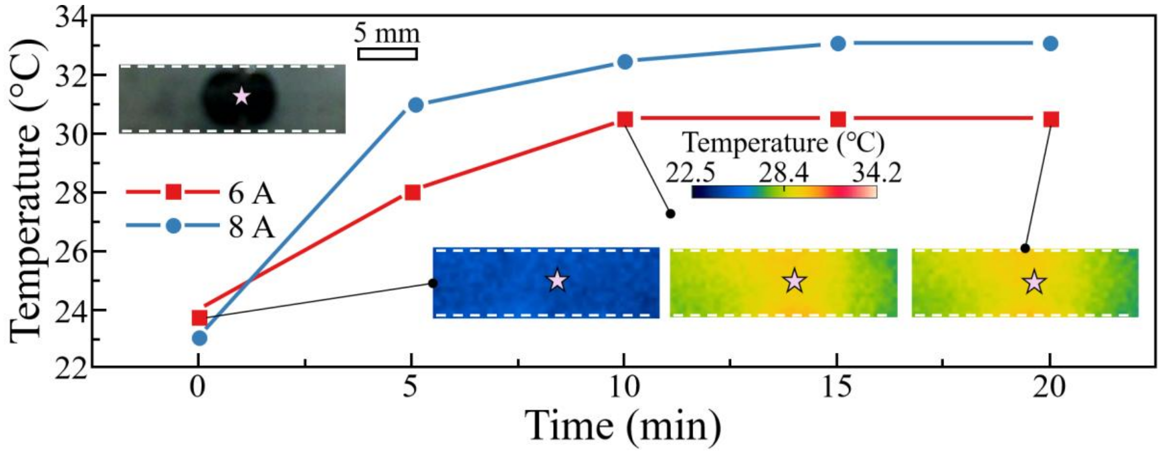

Figure S16. Demonstrating the increase and stabilization of temperature in the LMSS pump. Despite the high current used in our system, due to the extremely low resistance of the liquid metal, and the pump's inherent cyclic behaviour—which automatically creates an on-off duty cycle of approximately 50%—the temperature of the pump stays within a range safe for the human body. This allows for potential applications in wearable and implantable devices.

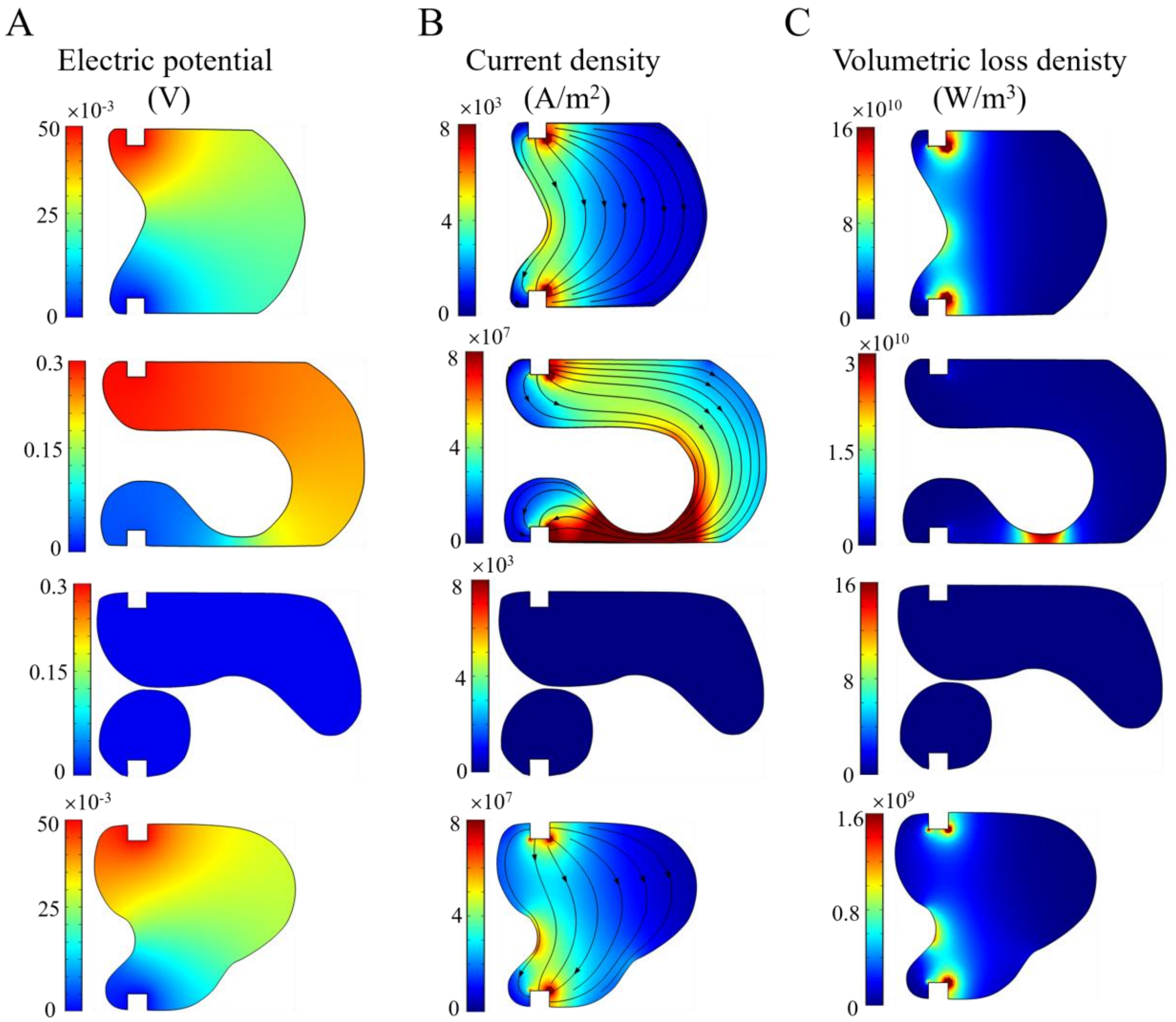

Figure S17. Finite Element simulations (COMSOL). A) Distribution of electric potential, B) Distribution of Current density, and C) Distribution of volumetric electric loss density, for 4 stages of the LMSS pump.

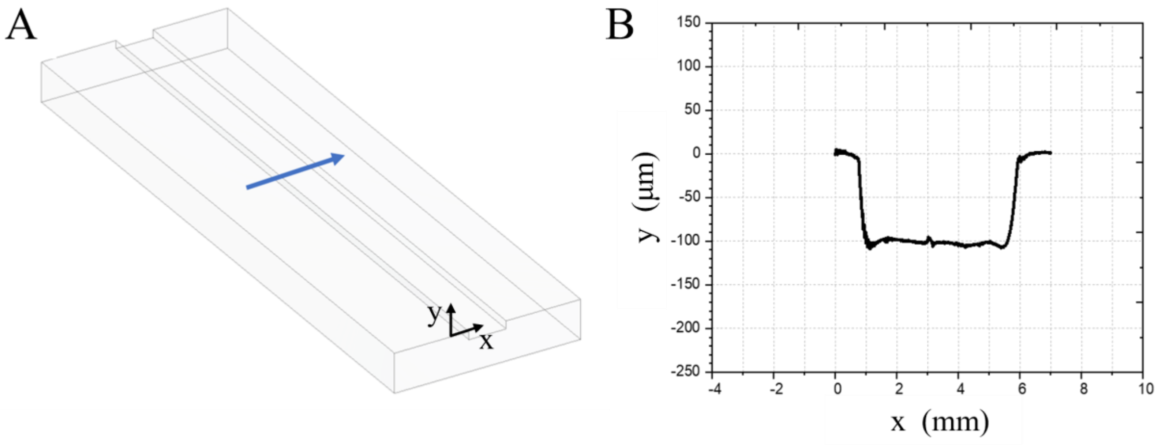

Figure S18. Characterisation of the PDMS microchannel. A) Path of the profilometer probe, B) Cross-section profile of the PDMS microchannel.

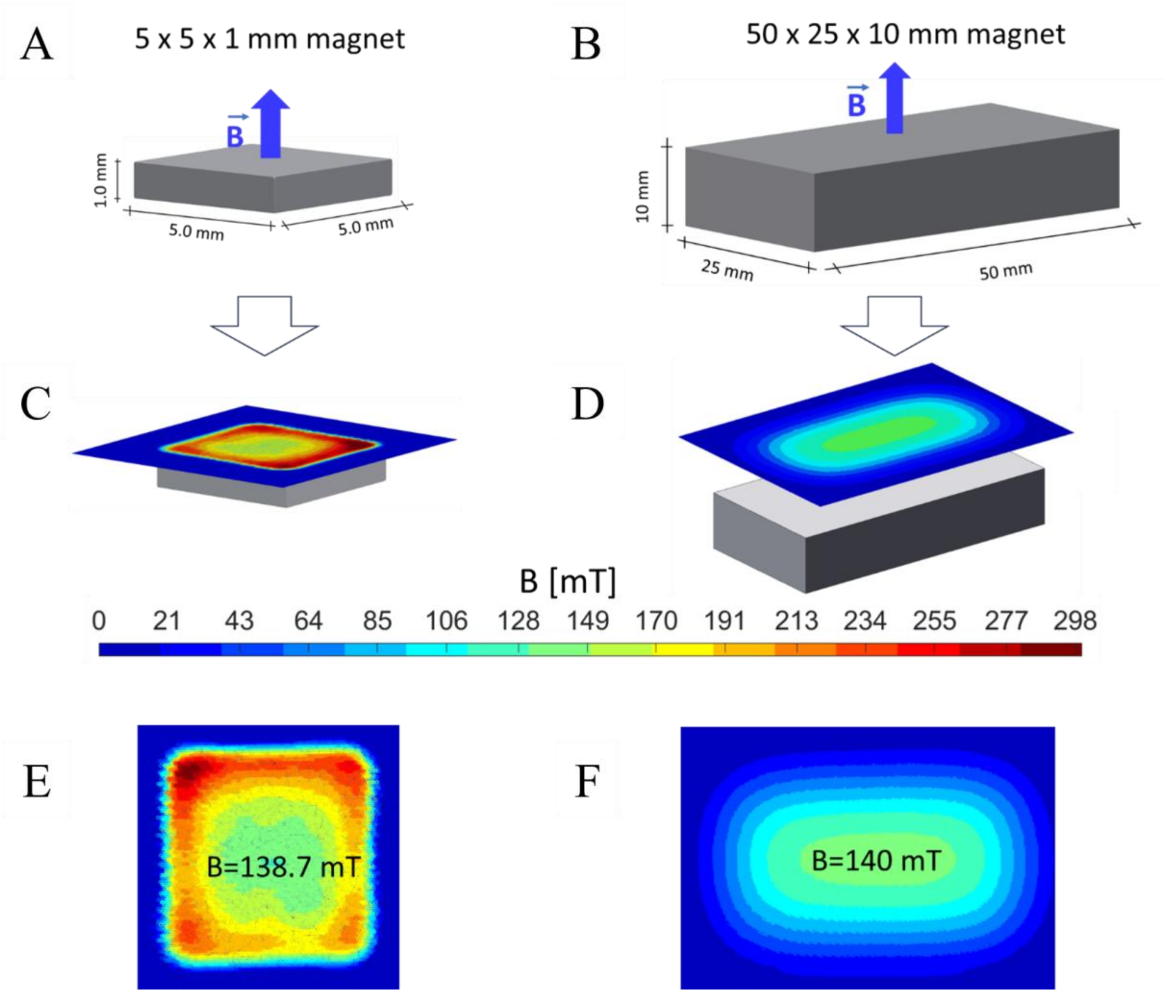

Figure S19. Magnetic field characterisation: A) small magnet. B) big magnet. C-D) Magnetic field characterisation of the magnets placed with the magnetic field facing upwards. E-F) 2D-profiles of the magnetic field intensity of the N52 magnets.

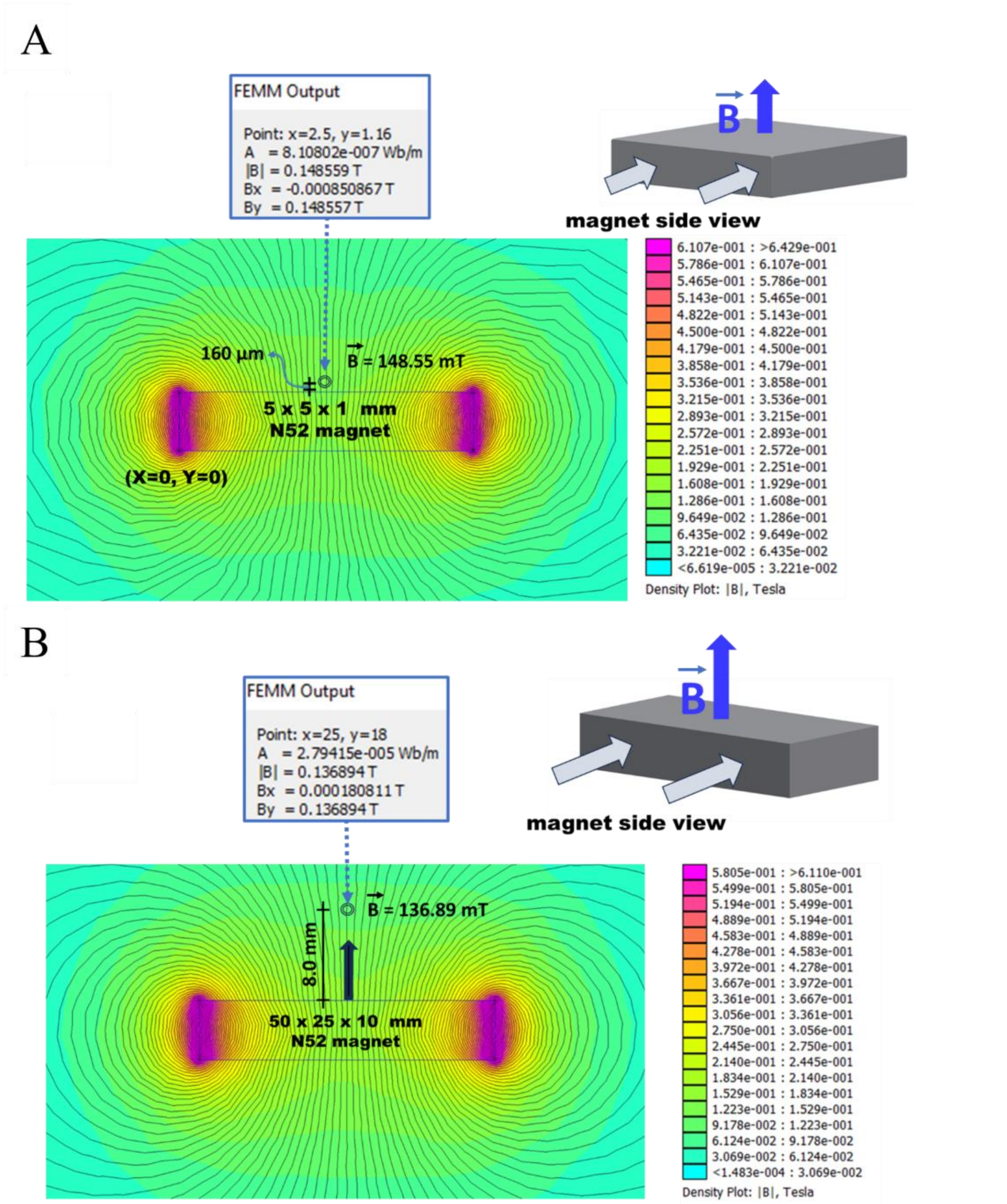

Figure S20. Magnetic field simulations. A) FEMM simulation of the small N52 magnet 5 mm × 5 mm × 5 mm. B) FEMM simulation of the big N52 magnet 50 mm × 25 mm × 10 mm. The magnetic field values measured at the magnet centres using both the gaussmeter and FEMM simulations are summarized in Table S4. These results highlight the effectiveness of both magnet configurations in generating comparable magnetic field strengths at the respective operating distances. The results shown in Table S4 correspond to the magnetic field value read in the centre of the surface of the magnets taken from the gauss meter and the FEMM simulations.

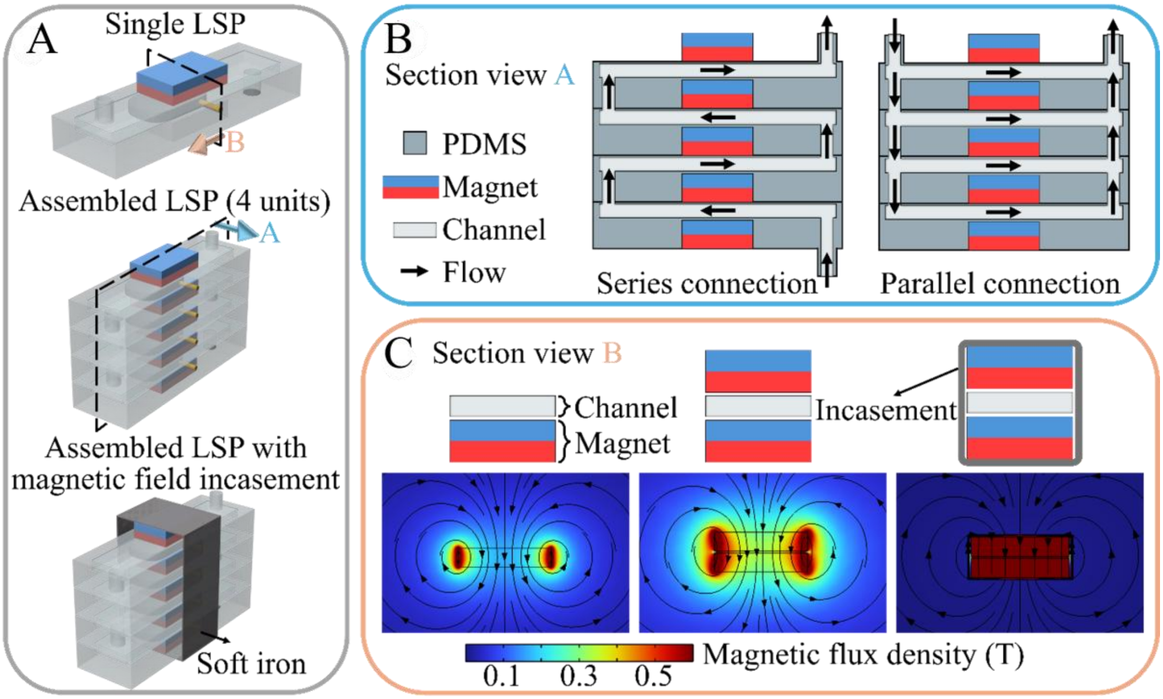

Figure S21. Scaling study. a) Diagram of scaling assembling structures of the LMSS pump. b) Possible internal connections for series and parallel configurations of the LMSS pump (the electrical connections are not shown). c) COMSOL study on the effect of the number of magnets and magnetic incasement. It can be seen that the magnetic flux density increases significantly by adding an incasement.

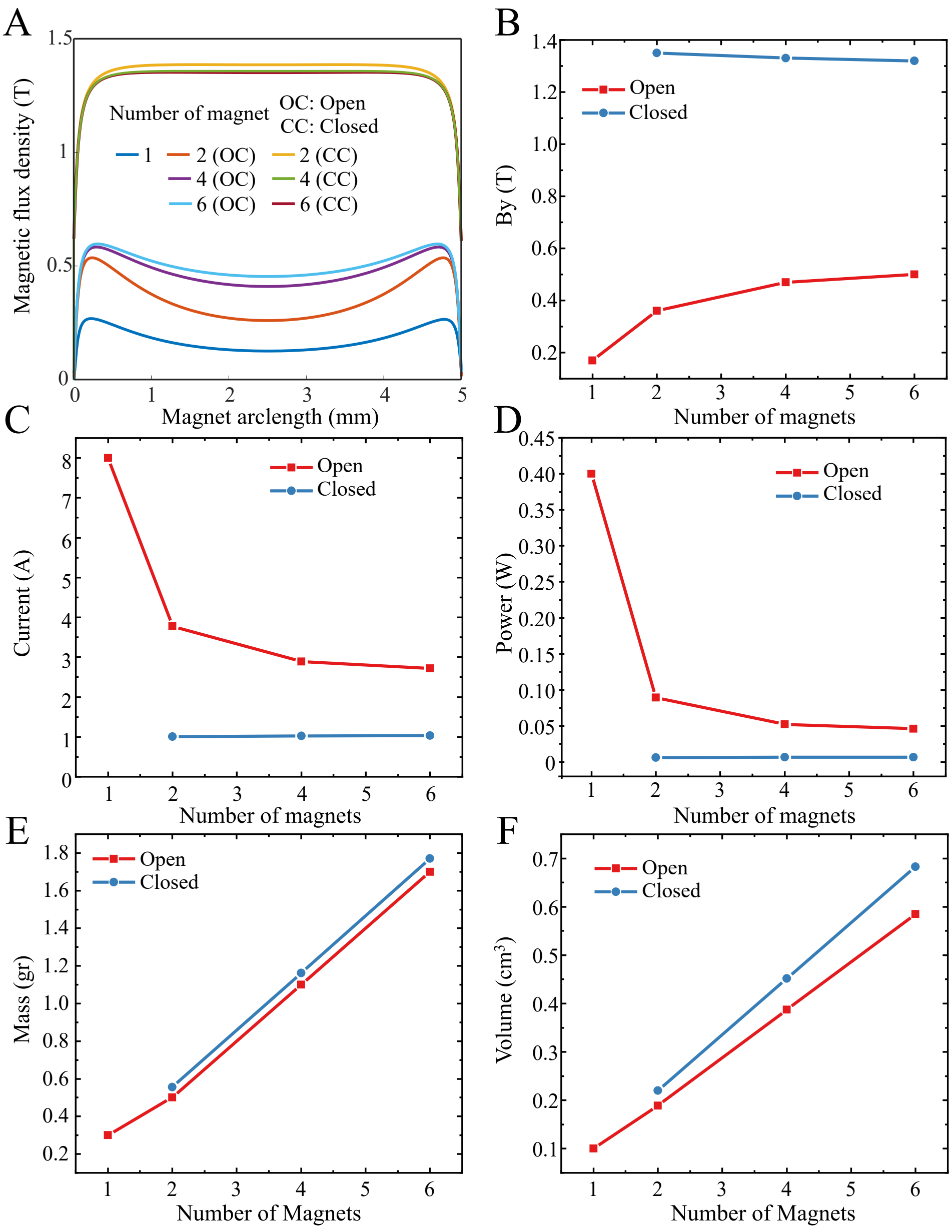

Figure S22. Demonstrating the effect of magnetic stacking and magnetic circuits on the LMSS pump characteristics. A) The magnetic flux density along a line 0.05 mm above the surface of the magnet (representing the middle of the microchannel). B) The integral of the y component of the magnetic flux density for different numbers of magnets. C, D) By increasing the magnetic flux density, the required current to achieve the same Lorentz force will be reduced, reducing power input to the LMSS pump. It can be observed that the input power can decrease up to 64 times using the magnetic circuit configuration. E, F) The change in mass and volume of the LMSS pump by scaling the number of channels and magnets.

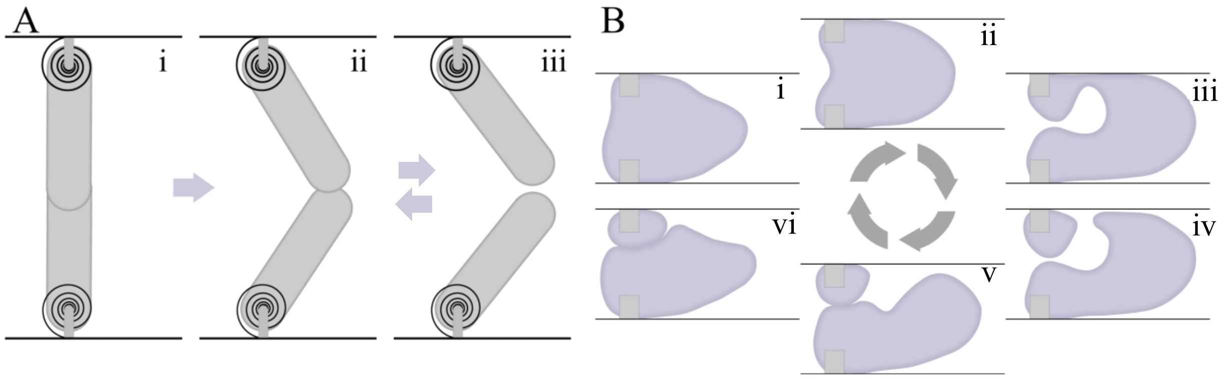

Figure S23. The necessity of breaking the symmetry to exploit hysteresis. A) A simple model of applying the Lorentz force to two solid conductors with sprung hinge fixings. The model will oscillate between the two states, ii and iii, and no fluid would be moved. B) The role of hysteretic power (i, ii, iii) and recovery (iv, v, vi) strokes in the LMSS pump.

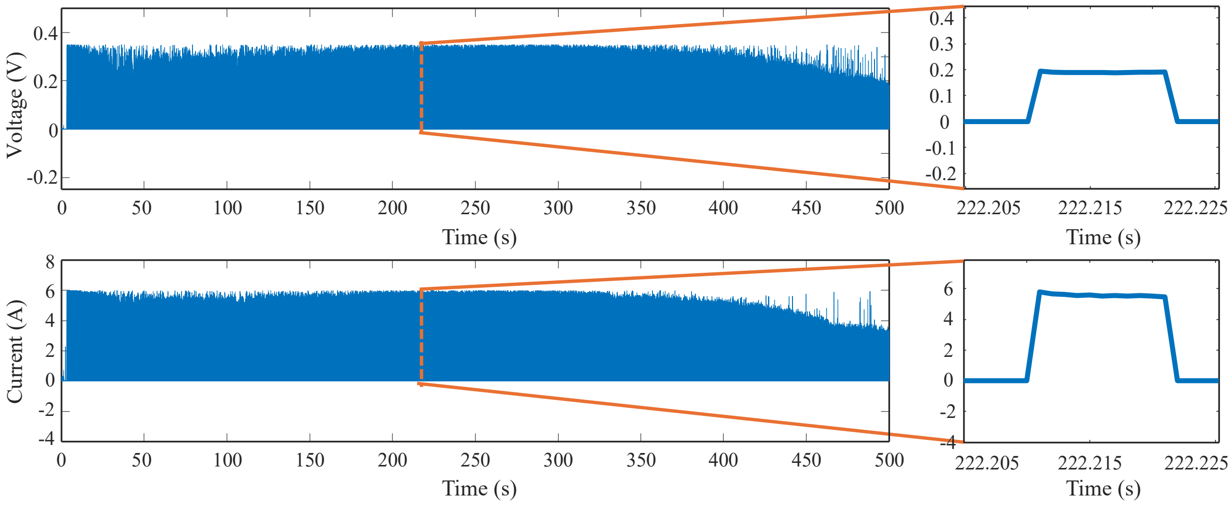

Figure S24. Voltage and current data acquisition of the LMSS pump powered by an AAA battery.

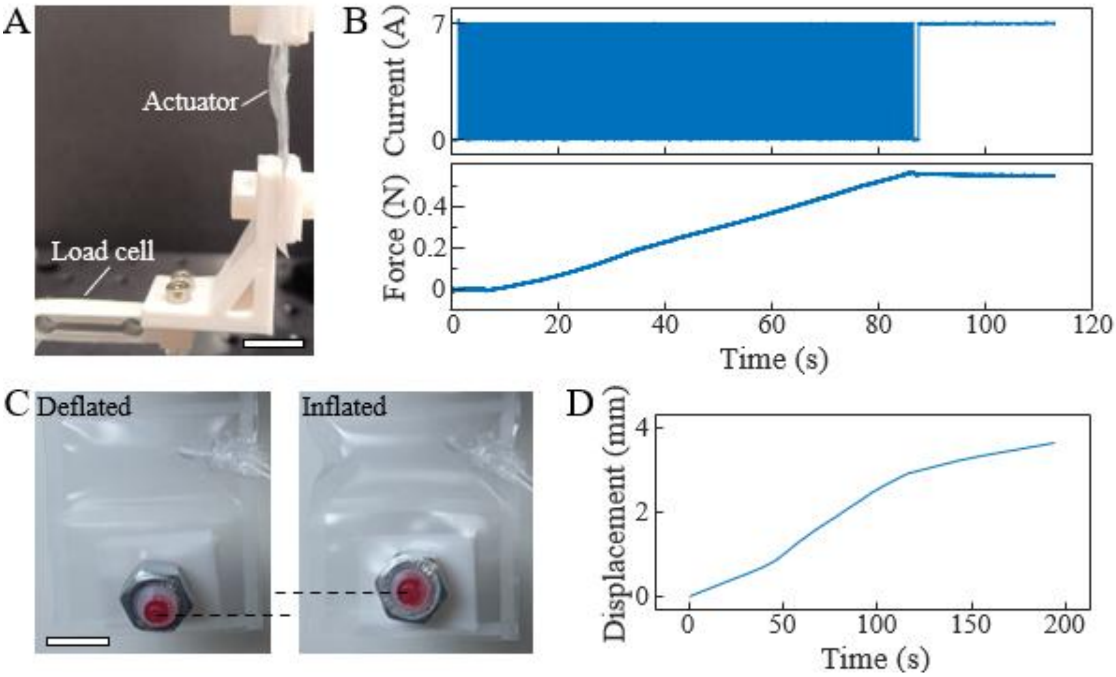

Figure S25. Linear pouch motor actuator driven by the LMSS pump. A) Set up for measuring the tension force generated by driving a single-pouch motor in linear mode using a load cell. B) The current applied to the LMS pump and the force generated against time. C-D) The linear actuator's displacement is measured when lifting a mass of 0.43 gr.

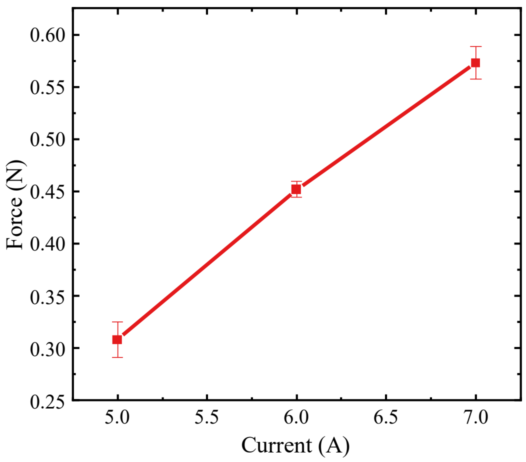

Figure S26. Isometric force measurements of the linear pouch motor for different currents applied to the LMSS pump.

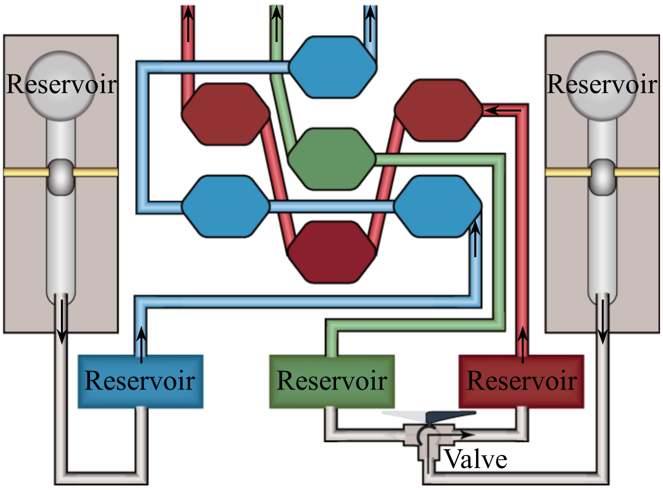

Figure S27. Schematic diagram of the soft fluidic display units and their connections to the two LMSS pumps. A three-way valve was utilized to facilitate the change in fluid colour, with manual switching performed at the desired time point. To evaluate the pumping performance of the LMSS pumps, the duration associated with manual valve switching was excluded from the measurement of colour display time. In future work, an automated valve-switching system will be developed to enable rapid and precise switching.

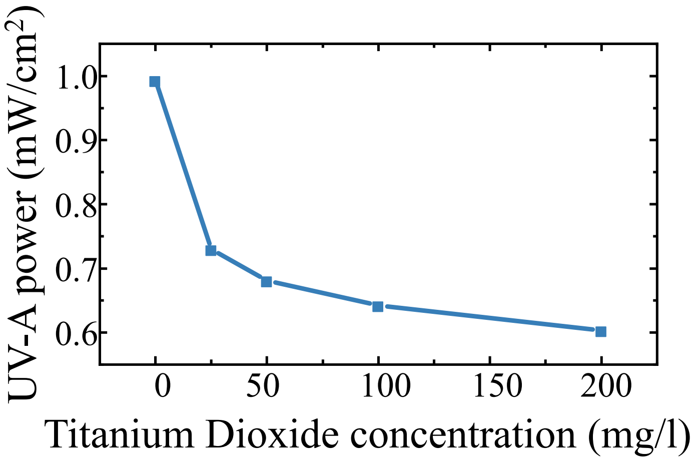

Figure S28. The relation of UV-A power detected with different concentrations of titanium dioxide pumped into the UV protection skin.

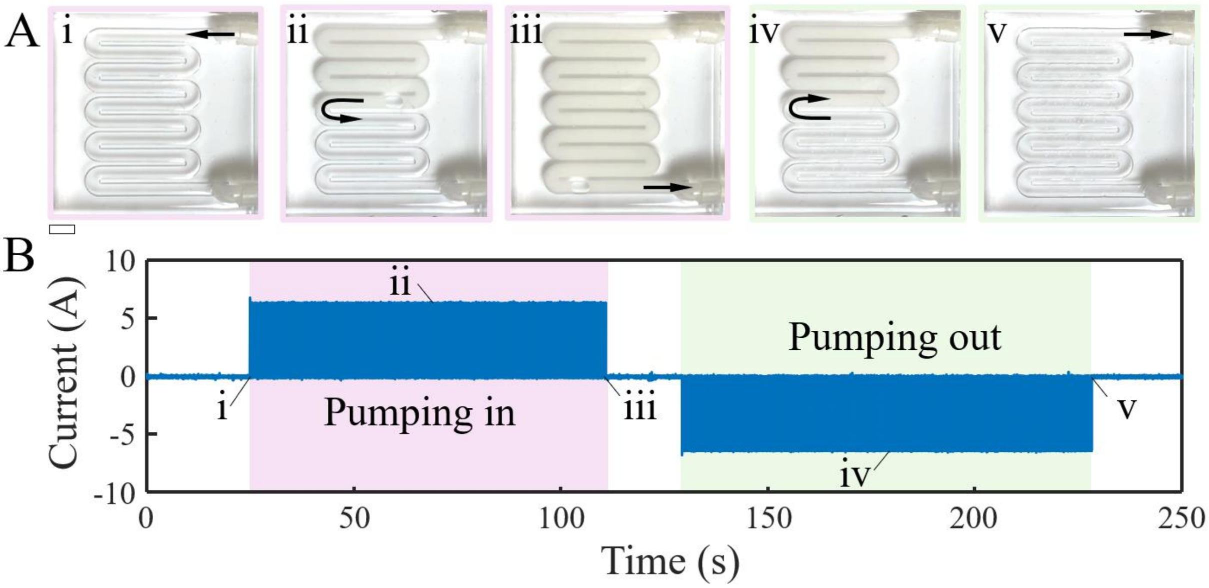

Figure S29. Demonstration of reversible flow for the UV protection skin utilizing the bidirectional capability of the LMSS pump. A) Shows pumping in (i-iii) and pumping out (iv-v) the opaque UV protection fluid. B) shows the current applied to generate bidirectional pumping. Scale bar is 10 mm.

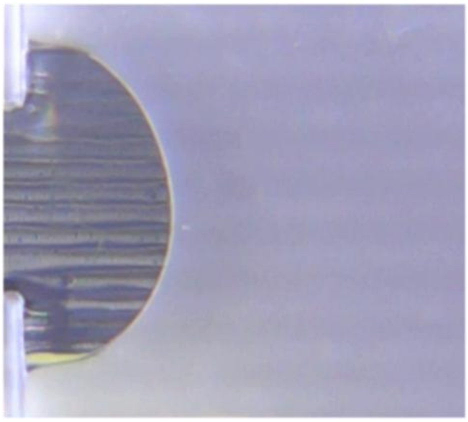

Figure S30. Top-down image of an injected EGaIn droplet inside a microchannel with a height of 0.1 mm. The measured contact angle of the EGaIn-NaOH (1M) interface was around 220°.

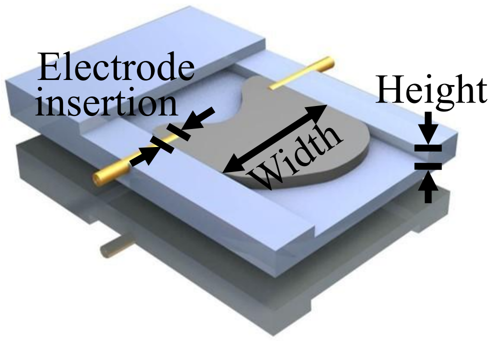

Figure S31. Pictorial definition of LMSS pump dimensions.

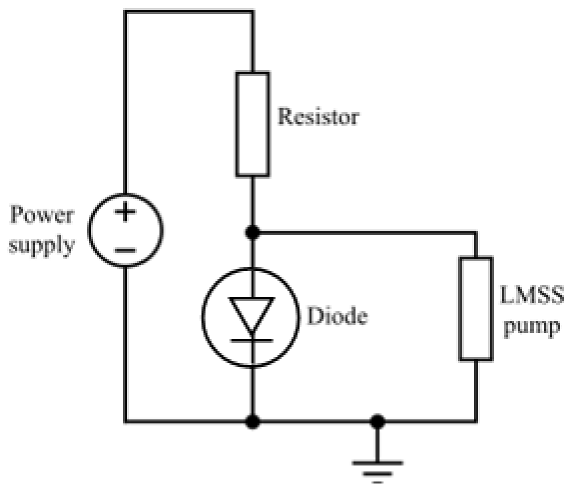

Figure S32. Simple voltage clamp using a diode. If the power supply powering the LMSS pump cannot be restricted to a specific voltage, a simple voltage clamping circuit using a silicone or Schottky diode can be utilised to prevent electrolysis in the LMSS pump channel. Schottky diodes have a forward voltage drop (V_f) typically in the range 0.15 V to 0.46 V, while for silicone diodes, V_f is typically around 0.7 V, all below the electrolysis potential required for water electrolysis (1.23 V). This circuit can be replaced by a microprocessor-controlled switch (e.g. MOSFET) to reduce the power consumption of the system.

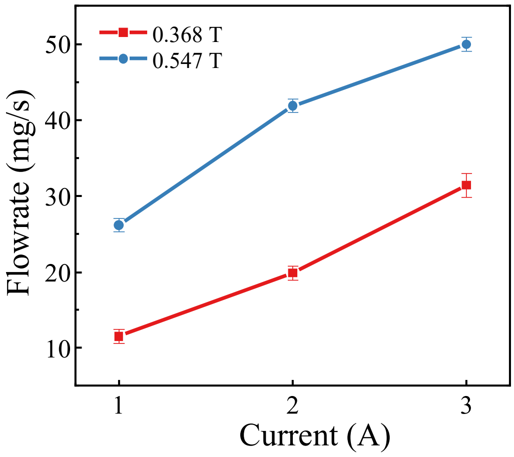

Figure S33. Demonstrating the flowrate at 1 kPa back pressure against current for increased magnetic field values. At a magnetic field of 0.368 T, we achieved the highest efficiency of 0.122 per cent, and at a magnetic field of 0.547 T, we achieved the highest efficiency of 0.22 per cent. This data supports our simulation results presented in Figure S22 and highlights the potential for further improving the efficiency of the LMSS pump by closing the magnetic circuit.

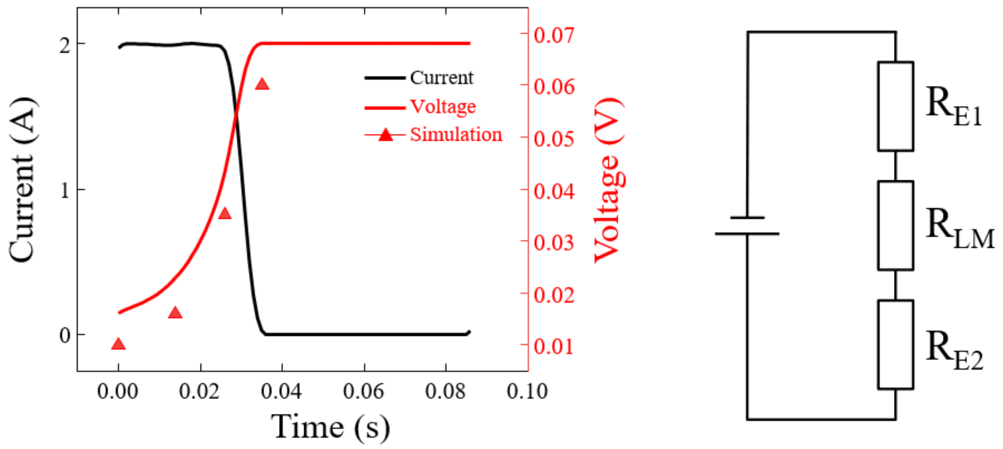

Figure S34. Left: Left: Demonstrating the voltage and current of a single cycle of the LMSS pump and the COMSOL simulated voltage (triangle markers) for the liquid metal droplet (R_{LM} for subset of deformation from Figure S17) without voltage loss at the electrodes (R_{E1} and R_{E2}). In our experimental work, there is a limit to how closely we can measure the droplet's voltage in situ; therefore, to determine the additional resistance introduced by wiring, we simulated the droplet at stages throughout the cycle and recorded the voltage at each and calculated the modelled efficiency as 0.35 per cent. Right: A simple diagram illustrating the circuit resistances in our experimental setup.

Table S1. Comparison of LMSS pump performance with leading hydraulic soft pumps and commercial pumps

Pump	Ref.	Mass (gr)	Vol. (cm^3)	Voltage (V)	Max power consumption (W)	Max pressure (kPa)	Max flowrate (ml/min)	Pressure/ Vol. (GPa/m^3)	Flowrate/ Vol. (kl/min m^3)	Pressure/ Vol. Power (GPa/m^3 W)	Flowrate/ Vol. Power (kl/min m^3 W)
						Soft pumps					
LMSS	This work	0.45	0.21	0.1	0.4	7	1.62	33.33	7.71	83.3	19.28
4 series		0.9	0.43	0.1	1.6	24		55.8		34.8	
EHD	*19*	1	1.17	10k	0.17	14	6	12	5.13	70.58	30.17
EHD	*18*	1.2	2.5	17k	0.71	80	45	32	18	45	25.35
DEA	*22*	13.5	15	1.7k	3.3	12.5	39	0.83	2.6	0.25	0.78
MHD	*38*	30	22.7	1	0.17	8	320	0.35	14	2	83
Magnetic	*35*	12.8	11.6	7.2	4	0.0014	134	0.0001	11.5	3E-5	2.8
						Commercial pumps					
MGD 1000S	*39*	142	58.6	24	30	800	500	13.7	8.5	0.45	0.28
McMaster STPAC	*40*	15422	75500	120	1200	1034	42500	0.01	0.56	8E-6	0.0004
Gotec ESX-04	*41*	35	24.6	230	5	30	83.3	1.2	3.38	0.24	0.67

Table S2. Simple model for the series and parallel configuration of LMSS pump. In the instances where LMSS pump units are arranged in series and parallel configurations, we introduce a basic model to demonstrate the potential for attaining increased pressures and flow rates within this system. By considering NaOH as an incompressible fluid, we can represent it as a highly stiff spring. Here, *d* represents distance, *F* denotes applied force, F_T represents the total applied force, *k* signifies spring stiffness, *V* represents volume, and *P* represents pressure.

		Series	Parallel
Diagram		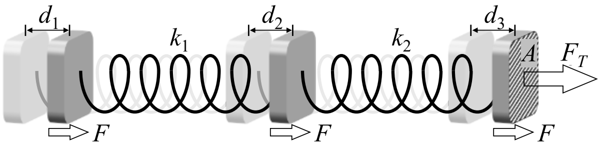	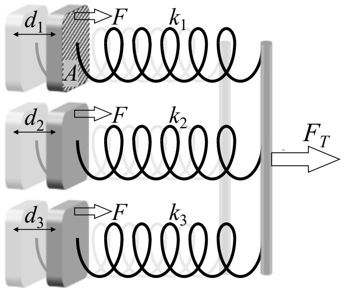
Condition (When synchronized)		$d_T = d_1 = d_2 = d_3 = d$	$d_T = d_1 + d_2 + d_3 = 3d$
		$F_T = F_1 + F_2 + F_3 = 3F$	$F_T = F_1 = F_2 = F_3 = F$
Results	Volume changed	$\Delta V = A \times d$	$\Delta V = A \times 3 \times d$
	Pressure	$P = 3 \times F \times A^{-1}$	$P = F \times A^{-1}$

Table S3. Profilometry depth of the PDMS microchannel

Sample	Microchannel depth (µm)
1	101.86 ± 2.5
2	109.22 ± 3.41
3	99.98 ± 8.02

Table S4: Results of the magnetic field characterisation and FEMM simulation. Results of the gauss meter readings and simulations: the magnetic field values are taken in the centre of the surface of the magnets. The distances from the surface of the magnet are 160 µm and 8 mm for the small and big magnet, respectively.

**Source: gauss meter / simulation**	**Magnet size (mm)**	**Height from the centre of the magnet's surface (mm)**	**Magnetic field intensity (mT)**
Gauss meter	5 × 5 × 1	0.160	138.7
Gauss meter	50 × 25 × 10	8.00	140.0
Simulation	5 × 5 × 1	0.160	148.5
Simulation	50 × 25 × 10	8.00	136.9

3 Supplementary Videos

Supplementary Video 1. Significant shape changes of unconstrained (explosive) and semi-constrained (unstable) liquid metal droplet driven by Lorentz force

Supplementary Video 2. LMSS in a constrained condition showing autonomous pumping

Supplementary Video 3. LMSS pump can be powered directly from AAA battery without a control circuit

Supplementary Video 4. Effect of different parameters on shape changes of liquid metal droplet

Supplementary Video 5. The hysteresis in liquid metal actuation generates power and recovery stroke

Supplementary Video 6. Comparison of the emerged shape changes between Lorentz force and syringe pump

Supplementary Video 7. Demonstrating the fluidic capabilities of LMSS pump

Supplementary Video 8. Demonstrating a compact, portable, battery-powered LMSS wrist pump for dynamic skin protection

Supplementary Video 9. The emergent self-synchronisation of multiple LMSS pumps in series

Supplementary Video 10. Demonstrating the bidirectionality of LMSS pump and its capability to pump air

Supplementary Video 11. LMSS pump operating under mechanical deformation at a curvature of 0.25 cm^{-1}

Supplementary Video 12. Demonstrating the dynamic adaptability of UV protection skin utilizing the bidirectionality of LMSS pump.
